# “The Dark Side of Musculoskeletal Care”: Why Do Ineffective Techniques Seem to Work? A Comprehensive Review of Complementary and Alternative Therapies

**DOI:** 10.3390/biomedicines13020392

**Published:** 2025-02-06

**Authors:** Lucas Mamud-Meroni, Germán E. Tarcaya, Andoni Carrasco-Uribarren, Giacomo Rossettini, Mar Flores-Cortes, Luis Ceballos-Laita

**Affiliations:** 1 Department of Kinesiology and Physiotherapy, Flores University, Neuquén Q8300, Argentina; lucas.mamud@uflouniversidad.edu.ar (L.M.-M.); german.tarcaya@uflouniversidad.edu.ar (G.E.T.); 2 Faculty of Medicine and Health Sciences, Universitat International de Catalunya, 08195 Barcelona, Spain; acarrasco@uic.es; 3 School of Physiotherapy, University of Verona, 37129 Verona, Italy; giacomo.rossettini@gmail.com; 4 Faculty of Health Sciences, Department of Physiotherapy, University of Malaga, 29071 Malaga, Spain; marflco@hotmail.com; 5 Department of Surgery, Ophthalmology, Otorhinolaryngology and Physiotherapy, University of Valladolid, 42004 Soria, Spain

**Keywords:** complementary therapies, musculoskeletal diseases, biological plausibility, placebo effect, evidence-based medicine, osteopathic medicine, chiropractic

## Abstract

The increasing interest in complementary and alternative medicines (CAMs) for musculoskeletal care has sparked significant debate, particularly regarding their biological plausibility and clinical effectiveness. This comprehensive review critically examines the use of two of the most widely utilized CAMs—osteopathy and chiropractic care—over the past 25 years, focusing on their biological plausibility, clinical effectiveness, and potential mechanisms of action. Our analysis of current research and clinical studies reveals that osteopathy and chiropractic are based on concepts such as “somatic dysfunction” and “vertebral subluxation”, which lack robust empirical validation. While these therapies are often presented as credible treatment options, studies evaluating their effectiveness frequently exhibit serious methodological flaws, providing insufficient empirical support for their recommendation as first-line treatments for musculoskeletal conditions. The effects and mechanisms underlying osteopathy and chiropractic remain poorly understood. However, placebo responses—mediated by the interaction of contextual, psychological, and non-specific factors—appear to play a significant role in observed outcomes. The integration of therapies with limited biological plausibility, whose effects may primarily rely on placebo effects, into healthcare systems raises important ethical dilemmas. This review highlights the need for rigorous adherence to scientific principles and calls for a more comprehensive investigation into biobehavioral, contextual, and psychosocial factors that interact with the specific effects of these interventions. Such efforts are essential to advancing our understanding of CAMs, enhancing clinical decision-making, promoting ethical practices, and guiding future research aimed at improving patient care in musculoskeletal disorders.

## 1. Introduction

Musculoskeletal care as a health discipline has seen significant advancements in recent years, largely driven by the adoption of evidence-based approaches. However, a concerning phenomenon persists: the continued use of therapies within musculoskeletal care that lack empirical support and even biological plausibility. In this sense, the use of complementary and alternative medicines (CAMs) has shown an increase in various regions worldwide, with notable variations. For instance, in Europe, their use has risen from 26% to 40% in recent years, with countries such as Germany and Denmark leading these statistics [1,2]. Similarly, in the United States, a comparable increase has been observed [3,4]. On the other hand, in Asia, CAMs enjoy significant social and institutional acceptance, which fosters an even greater inclination toward their use, particularly in countries such as China, the Philippines, and South Korea [5].

Despite efforts by part of the healthcare community to promote evidence-based practices, the previous statistics showed that contemporary musculoskeletal care remains influenced by the historical adoption of CAMs that lack scientific backing. This phenomenon has gained traction despite the lack of empirical evidence due to the growing demand from patients for more integrative and holistic approaches, often as a response to dissatisfaction with conventional medicine and its associated adverse effects [6,7]. However, the popularity of a therapy is a poor indicator of its effectiveness, and interventions must demonstrate their true value through methodologically rigorous studies.

The most described negative experiences with conventional medicine range from unfavorable interactions between professionals and patients to perceptions of inefficacy and the side effects of traditional treatments [8]. However, the rejection of conventional medicine or poor perception of healthcare systems are not the only factors driving the use of CAMs. On the contrary, users of these therapies often are proactive individuals who choose their own treatments, seeking approaches they consider most effective [9]. Additionally, many of these practices, seemingly grounded in science, are used by healthcare professionals, which can generate greater confidence in their adoption by the public [8].

To illustrate this situation, consider pharmacological treatments for headaches, which are typically the first line of intervention for patients [10,11]. However, these medications are not without risks, and approximately one-third of headache patients report dissatisfaction with the results [12]. Consequently, many turn to CAMs, such as chiropractic care [13,14] or osteopathy [15].

CAMs are defined as “a diverse set of medical and healthcare systems, practices, and products that are not currently considered part of conventional medicine” [16]. They are characterized by a limited number of clinical trials supporting their hypotheses or testing methods to evaluate their results in diverse conditions, as they were originally considered outside of evidence-based practices. However, this situation has evolved, and numerous journals specializing in complementary and alternative therapies have been established and categorized within the Journal Citation Reports of the Web of Science database under Integrative and Complementary Medicine [17].

A clear distinction between complementary and alternative interventions must be pointed out. Complementary therapies, on the one hand, accept various models of disease and are open to being used alongside evidence-based conventional therapies. Alternative therapies, on the other hand, propose a unique model of disease that attempts to explain the entire complexity of health and illness [18]. Examples related to musculoskeletal care include osteopathy with its “law of the artery” and chiropractic care with its “law of the nerve” [19]. These types of therapies often resist verification and falsity, where verification is understood as the biological plausibility of their hypotheses and falsity as admitting that if their effects are not superior to a placebo, they cannot be considered evidence-based techniques [20,21].

The classification of these CAMs indicates that none have been subjected to rigorously designed and executed scientific studies that address essential questions such as their biological plausibility or clinical effectiveness. In contrast, when these therapies respond favorably to the scientific method, they are removed from the lists of CAMs and integrated into the repertoire of evidence-based techniques [22]. The importance of this discussion lies in the fact that in the best-case scenario, CAMs may not have a direct adverse effect. However, for many patients, they can be counterproductive by significantly delaying appropriate treatment and preventing or interfering with access to quality healthcare based on the scientific method [23].

**Figure 1:**
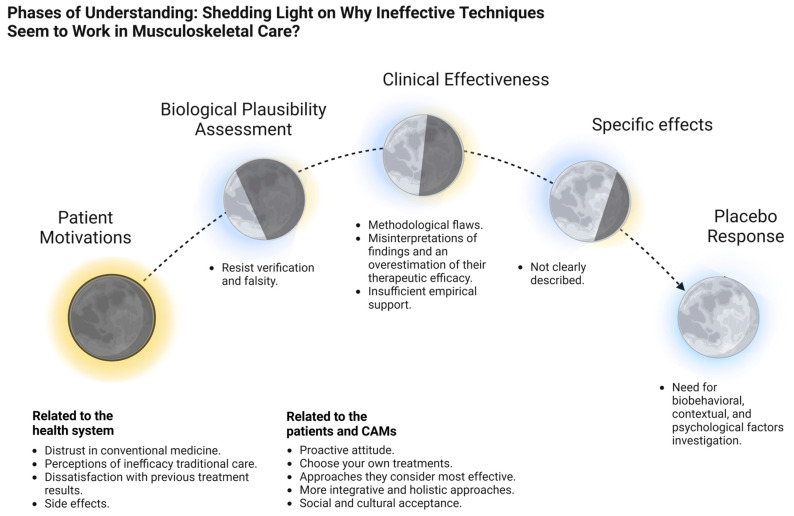
Phases of understanding: shedding light on why ineffective techniques seem to work in musculoskeletal care. This graphical abstract illustrates the progressive phases explaining why some ineffective techniques seem effective in musculoskeletal care. Represented by lunar phases, the graphic symbolizes the transition from the darkness of our current knowledge to the light this review aims to shed. Each phase progressively illuminates key elements involved in the use of CAMs—osteopathy and chiropractic—in musculoskeletal care, bringing clarity to what was previously obscure. Created in BioRender. F, M. (2025) https://BioRender.com/g83d974, accessed on 26 January 2025.

In this context, there is a growing need for a critical comprehensive review of CAMs used in musculoskeletal care, focusing on their biological plausibility, clinical effectiveness, and the factors involved in clinical responses and effects, including potential mechanisms of action, contextual factors, placebo response, and psychological influences such as cognitive biases (Figure 1).

This review aims to examine these key dimensions while maintaining a balanced perspective, offering practical implications for both clinical practice and future research. Specifically, it is intended to provide guidance on the appropriate integration of CAMs as a first line for musculoskeletal care. Furthermore, the review highlights the necessity for robust, high-quality studies to address existing gaps in evidence, fostering open and constructive dialogue among healthcare professionals. This balanced approach is intended to support evidence-based decision-making, promote ethical clinical practices, and encourage the advancement of integrative healthcare research.

## 2. Methodology of Literature Search

This critical comprehensive review was conducted following a different search strategy to identify relevant literature on CAMs used in musculoskeletal care [24]. The search was performed in multiple databases, including PubMed, Scopus, Web of Science, and Cochrane Library. The search was limited to articles published in English, spanning from January 2000 to January 2025. A manual search of reference lists from included articles was also conducted to ensure that no significant studies were omitted.

### Eligibility Criteria

Study selection for this narrative review followed predefined inclusion and exclusion criteria to ensure methodological rigor and alignment with the research objectives.

Eligible studies were those investigating the biological plausibility, underlying mechanisms of action, or clinical efficacy of CAMs, specifically osteopathy and chiropractic, in musculoskeletal care. The study types included narrative reviews, systematic reviews, meta-analyses, original research articles, theoretical papers, and opinion/commentary articles. This broad scope allowed for the generation of hypotheses regarding the mechanisms underlying the use of CAM in musculoskeletal health. The comprehensive approach adopted in this review facilitated the inclusion of diverse sources, enriching the understanding of CAM’s role in musculoskeletal care.

Studies were excluded if they focused on non-musculoskeletal conditions or primarily addressed psychological or wellness outcomes that were not directly relevant to musculoskeletal care. However, studies examining psychological effects (e.g., biases, anxiety, depression) as part of a broader analysis of CAM interventions in musculoskeletal conditions were included when they provided meaningful insights into the overall impact on patient well-being.

## 3. Biological Plausibility of CAMs

The discussion surrounding the biological plausibility of osteopathy and chiropractic in musculoskeletal care is a crucial aspect that demands careful analysis. Understanding the challenges inherent in the theoretical models underpinning these therapies is essential for evaluating their validity.

According to Koterov [25], biological plausibility serves as a fundamental pillar for establishing causal relationships in epidemiological research and health sciences. The concept rests on the premise that a causal link should conform to the prevailing scientific theories and align with the body of biological knowledge available. For an association to be deemed causal, it is essential to identify a coherent biological pathway that clarifies how one factor might influence another. In the absence of such a model, it is difficult to definitively confirm causality. This becomes especially critical in public health, where decisions regarding prevention and safety standards hinge on the validity of the scientific basis supporting the therapies being recommended or implemented [25].

### 3.1. Biological Plausibility of Osteopathy

Osteopathy was founded by the American physician Andrew T. Still, and claims that “somatic dysfunctions” in the musculoskeletal system are linked to both musculoskeletal and non-musculoskeletal conditions, considering osteopathic manipulative treatment as the main intervention to treat these dysfunctions. Somatic dysfunctions can affect the skeletal, vascular, and neural systems and can originate pain, organ dysfunction, or impaired systemic health, highlighting the interconnection between the musculoskeletal system and other body systems [26]. This approach suggests that osteopathic manipulative treatment can address a wide array of clinical conditions, from substance abuse to musculoskeletal pain. However, this broad application risks undermining the scientific credibility of osteopathic practice, potentially leading practitioners to disregard established scientific consensus and perpetuate epistemological gaps in their practice [27].

Osteopathy is based mainly on three large groups of treatments or interventions: structural osteopathy, craniosacral osteopathy, and visceral osteopathy.

Structural osteopathy places emphasis on the interconnectedness of body systems and the body’s inherent self-healing capabilities. Dysfunctions within the musculoskeletal system may affect visceral organs via somatovisceral reflexes, while visceral pathologies may present as restricted movement or modification in the tissue consistency in the musculoskeletal system, termed viscerosomatic reflexes [26]. Osteopathic manipulative treatment aims to correct these dysfunctions by relieving pain and improving range of motion, as well as improving neurovascular and lymphatic flow [26,28]. However, the efficacy of this approach is highly dependent on the identification of “somatic dysfunctions”, a process with limited inter-rater reliability between practitioners [29].

Craniosacral osteopathy is based on the premise that alterations in the mobility of cranial sutures can cause diseases, disorders, or dysfunctions. It is argued that a disruption in the “mobility” of the sphenobasilar synchondrosis could lead to disturbances throughout the cranial complex, a diagnosis typically made through specific palpatory assessment by an osteopath. This approach often employs general concepts of proven biological phenomena to infer that similar processes occur within the osteopathic model. For instance, rhythmic movements observed from the cellular level to the cardiac level are used to suggest that the craniosacral system and its surrounding structures must exhibit a similar oscillatory rhythm [30]. However, it is noteworthy that findings from magnetic resonance imaging (MRI), which suggest that the skull might exhibit dimensional variations of 0.898 mm/pixel (less than a millimeter) [31], are often dismissed due to potential interpretation errors [30]. These results could stem from vestibular system stimulation, generating rhythmic head movements. Nevertheless, there is skepticism about the ability of manual therapists to perceive these submillimetric changes with their hands, as such variations fall outside the resolution capacity of MRI [31,32].

Finally visceral osteopathy proposes that manual manipulation can improve the mobility of internal organs and restore their function, based on the idea of interconnection between visceral and musculoskeletal structures [33]. However, studies investigating this supposed visceral mobility through osteopathic techniques have yielded inconsistent and inconclusive results. Moreover, the biomechanical and physiological principles supporting this notion lack grounding in human anatomy and physiology. While it is observed that viscera exhibit mobility during vital functions such as breathing and activities like running and jumping [34], a clear causal relationship between the alleged alteration of visceral mobility in various clinical conditions and the manual manipulations intended to restore it has not been established. Furthermore, osteopathy often falls into the fallacy of anatomical possibilism, exaggerating anatomical-functional relationships to the point of implausibility [35]. Additionally, the reliability of diagnostic techniques used in visceral osteopathy lacks solid evidence, and there is no consensus on the existence of “somatic dysfunction” [36,37].

### 3.2. Biological Plausibility of Chiropractic

Chiropractic was founded by David Palmer and is mainly based on the theory of “vertebral subluxation”, which claims that misaligned vertebrae can cause interference in the nervous system, subsequently affecting the function of other bodily systems, such as the immune system, and contributing to the development of diseases [38,39]. However, this theory has sparked intense debate and controversy within the scientific community due to the lack of robust evidence supporting its biological plausibility [40,41]. Moreover, the treatment for this proposed complex and multisystemic dysfunction involves spinal manipulation or chiropractic adjustment, which many consider an effective tool for relieving ailments and improving neurological function [42]. Proponents of chiropractic argue that these are specific assessments and maneuvers aimed at correcting vertebral subluxations. Nevertheless, studies have shown that segmental vertebral evaluation and manipulations cannot be specifically applied to a particular vertebra and that their limited effects are based on nonspecific mechanisms [43].

In the discussion of new causal mechanisms, two general errors or biases can occur, identified as “the believers and the skeptics” [44]. The first error, the believer’s error, is inferring the existence of a causal mechanism when it does not exist where osteopathy, chiropractic care, and other CAMs may fall. The second, the skeptic’s error, is inferring the nonexistence of a causal mechanism that exists, an error we must strive to avoid until evidence suggests otherwise. In this sense, osteopathy and chiropractic describe a causal mechanism that is not supported by biological bases; therefore, its effects may rely on non-specific, placebo, and contextual factors [45,46]. On the other hand, conventional interventions such as exercise have described multiple mechanisms of action with strong biological bases, specific effects and long-term effects.

The reviewed articles encompass a wide range of study types, including observational studies, reviews (narrative, systematic, and conceptual), and educational model validations. Interventions analyzed focus primarily on osteopathy, chiropractic care, visceral mobilization, and spinal manipulation. Populations studied ranges from the general public to patients with spinal pain, pediatric groups, and osteopathy students. Key outcomes include assessments of clinical efficacy, biological plausibility, and theoretical mechanisms. However, the studies are frequently limited by theoretical frameworks, small sample sizes, and a lack of robust experimental evidence. The reader can find the studies analyzed in Appendix A.

## 4. Clinical Effectiveness of Osteopathy and Chiropractic in Musculoskeletal Care

### 4.1. Clinical Effectiveness of Osteopathy

Osteopathy, encompassing structural, visceral and craniosacral techniques, has been subject of debate regarding its efficacy in musculoskeletal care. Although the effectiveness of pragmatic osteopathy has been studied in some systematic reviews with meta-analyses [47,48,49,50,51], and associated with small statistical effects on clinical outcomes, the methodological flaws identified cast doubt on the positive results. The most important biases found were the inclusion of congress abstracts, pilot studies that do not aim to evaluate clinical effectiveness, and unpublished materials from osteopathic institutions as relevant studies. Recent systematic reviews with meta-analyses with more robust methodological approaches found that pragmatic osteopathic manipulative treatments, and visceral and craniosacral osteopathy in isolation produced no statistically significant effects on clinical outcomes on patients with musculoskeletal or non-musculoskeletal disorders [36,52,53,54,55,56,57,58]. Specifically, a recent systematic review and meta-analysis demonstrated that the pragmatic application of osteopathic manipulative treatment was not superior to sham or placebo interventions for patients with neck and low back pain. This finding suggests that the effects of osteopathy may be attributed more to placebo effects than to specific therapeutic mechanisms [29].

Several critical methodological issues and biases have been identified in primary clinical trials. One of the most significant concerns is the diagnosis based on the manual palpation of so-called somatic dysfunctions, whose reliability has been shown to be questionable due to poor inter-examiner agreement and the inability to standardize this method. Additionally, there is a lack of standardization in the parameters of the techniques employed, such as the applied force, the duration of the interventions, and the pragmatic application based on palpatory findings.

Other relevant factors include the use of placebo techniques with questionable efficacy, the absence of proper evaluation of masking effectiveness, the use of small sample sizes, and the presence of groups with high variability and elevated standard deviations. These methodological shortcomings significantly undermine the robustness and validity of the results obtained [56,59,60,61,62].

### 4.2. Clinical Effectiveness of Chiropractic

Chiropractic care faces similar scrutiny regarding its clinical effectiveness. It is often criticized for lacking a plausible biological model for its interventions. While short-term improvements in spinal symptoms are modest, these often lack clinical significance [63,64]. Moreover, chiropractic interventions have not demonstrated superiority over other treatments, and their effectiveness compared to placebo or no intervention remains questionable [65,66]. The use of chiropractic care for conditions unrelated to the spine is not supported by evidence [67].

Spinal manipulation is the cornerstone of chiropractic care and has been associated with frequent mild adverse effects and, in rare cases, severe complications of unknown incidence [68]. Chiropractors appear to have the highest number of adverse events following manipulations among healthcare professionals [69], while the low quality of evidence regarding sham manipulation introduces further uncertainty in comparisons between interventions.

The reader can find the studies analyzed in Appendix B.

## 5. Effects and Potential Mechanisms of Osteopathy and Chiropractic in Musculoskeletal Pain

**Figure 2:**
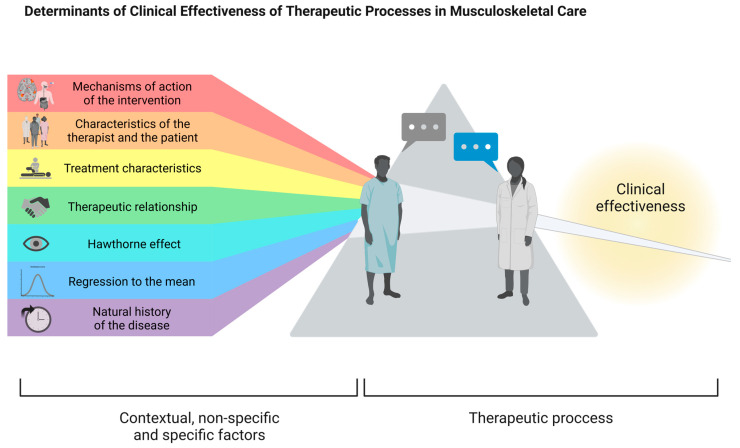
Determinants of the clinical effectiveness of therapeutic processes in musculoskeletal care. The clinical effectiveness of a therapy extends beyond the results of controlled studies, as it is influenced by a complex interaction between the specific effects of a therapeutic intervention and contextual and non-specific factors [45,46]. Non-specific effects include the natural history of the disease, fluctuations in symptom severity, regression to the mean, measurement errors, or the Hawthorne effect; while contextual factors include characteristics of the therapist and patient, the relationship between them, characteristics of the treatment, and the healthcare setting. These are just a few examples, but many more factors can be considered. Illustration adapted from Hohenschurz-Schmidt et al. [70]. Created in BioRender. F, M. (2025) https://BioRender.com/g83d974, accessed on 26 January 2025.

The overall treatment effect is divided into three interacting factors: specific, contextual, and non-specific. Specific effects are clinical changes due to the direct mechanisms of the intervention. Contextual effects, on the other hand, result from the therapeutic encounter and the context of the health care system. Finally, non-specific effects, such as the natural course of the disease or regression to the mean, influence the clinical response but are not related to the intervention. The sum of contextual and non-specific effects is called the placebo response, based on changes perceived by the patient independently of the specific effects [45,46]. It is important to clarify that these factors influence each other and cannot be completely isolated (Figure 2).

### 5.1. Specific Factors in Osteopathic and Chiropractic Practices for Musculoskeletal Care

Specific factors for osteopathy and chiropractic have not been clearly demonstrated yet. Spinal manipulation therapy (SMT) is probably the most investigated technique that has shown some biological plausibility and is a common technique used by both osteopaths and chiropractors.

SMT is proposed to alleviate musculoskeletal pain through various neurophysiological mechanisms. At the peripheral level, the spinal manipulation may reduce proinflammatory cytokine activity and oxidative stress, potentially mitigating inflammation and peripheral sensitization [66]. At the spinal level, SMT is believed to induce segmental inhibition, which decreases temporal summation of nociceptive signals, thereby dampening central sensitization processes [71,72]. These effects have been observed in increased pain pressure thresholds in corresponding dermatomes and myotomes, as well as reduced sensitivity to thermal stimuli [73].

However, while SMT influences these neurophysiological parameters, these changes often fail to correlate consistently with improvements in pain, stiffness, or functional outcomes [74]. Furthermore, descending inhibitory pathways may contribute to reduced pain perception, but it remains unclear whether this is a specific result of SMT or attributable to non-specific or contextual factors [75]. It has been proposed that efforts are needed to improve the quality of studies and methods in order to know whether there are specific effects of these interventions and what their real contribution is to the overall effect of clinical outcomes [76].

### 5.2. Placebo Response: Interaction Between Non-Specific and Contextual Factors in Osteopathic and Chiropractic Practices for Musculoskeletal Care

The placebo and nocebo effects are therapeutic responses that arise independently of the intrinsic efficacy of an intervention. Specifically, the nervous system determines whether a treatment response is perceived as favorable or not [77,78,79]. These phenomena, which can be either positive or negative, are rooted in behavioral, emotional, and cognitive modulation [78,79].

The placebo response encompasses a wide range of factors, including personality traits [80,81], the patient–therapist relationship [82,83], cultural influences [84,85], genetics [78], conditioned responses [86], observational learning [87], descending modulation mechanisms [88], and brain dynamics [79,89]. These factors seem to contribute significantly to the outcomes observed in CAMs [90]

The placebo response is inseparable from clinical practice and is triggered by the sum of the interaction of contextual and non-specific factors. However, it is important to note that relying on therapies that depend solely on the manipulation of these factors leads to clinical responses with great variability and unpredictability [91,92]. Furthermore, the long-term stability of these effects is uncertain, suggesting that they may be of limited value and may interfere with appropriate treatments [93]. CAMs may also generate a nocebo response, which could worsen the patient’s condition.

Contextual and psychological factors are fundamental to the placebo phenomenon [84,94]. Therapies such as osteopathy or chiropractic often leverage these elements, intentionally or not [95]. For instance, in conservative, primarily passive treatments for patients with non-specific chronic low back pain, about half of the overall treatment effect can be attributed to non-specific effects that occur without any treatment rather than to specific effects or placebo effects induced by the therapies [96].

It is important to recognize that the placebo response represents only one facet of the numerous variables influencing the interaction between a patient, therapist, and clinical situation. The outcomes of any given intervention can be affected by non-specific effects, such as the natural history of the disease, regression to the mean [46], or the expectations generated by the therapy [97].

### 5.3. Cognitive-Mediated Effects and Bias in Osteopathic and Chiropractic Practices for Musculoskeletal Care

Emotional, cognitive, and social factors play a pivotal role in how individuals respond to therapy. The attention, care, emotional support, and explanations provided during treatment can significantly influence the patient’s experience, irrespective of the treatment’s actual efficacy [90].

The meaning attributed to a symptom and the approach taken towards it are crucial in determining the therapeutic response. For example, postoperative pain, which may be perceived as part of the healing process, can be experienced and tolerated differently compared to pain associated with a terminal illness, which is often linked to death and a palliative approach [98]. Some CAMs offer alternative narratives that allow patients to reinterpret their experiences, thereby enhancing their psychological well-being. Participation in therapy groups or communities that support these practices can provide a sense of belonging and social support, which contributes to emotional well-being [99,100,101].

These psychological responses are further influenced by cognitive biases defined as systematic patterns that can distort perception, memory, and reasoning and lead to erroneous conclusions or suboptimal decisions [102]. Such biases can arise from altered information processing [103], past experiences [104], personal beliefs and learning about health, illness, and care processes [102,105] as well as social and cultural influences [102].

The literature highlights several cognitive biases, such as causality bias and authority bias, that appear to play an important role in the therapeutic context. Furthermore, biases such as optimism bias, illusion of control bias, and confirmation bias may also influence the dynamic between patient and therapist, potentially shaping treatment perceptions and outcomes.

Causality bias occurs when a specific outcome is mistakenly attributed to a particular action, often due to a low demand for evidence [103,105]. Individuals prone to this bias tend to attribute any change in their health to a practice that aligns with their beliefs or previous experiences [106].

Authority bias occurs when the opinion of an expert, such as a health professional, is deemed sufficient for decision-making by the patient [107]. Research has shown that many health practices not supported by scientific evidence are recommended by professionals, who may themselves have limited understanding of the practice they endorse [108,109,110].

Optimism bias reflects the tendency to overestimate positive outcomes and underestimate potential risks [111], which can lead patients to believe that these therapies are inherently safe or more effective than the evidence suggests.

Illusion of control bias refers to the belief that one can influence outcomes beyond actual control; this can reinforce both patient and practitioner confidence in these interventions, even when objective evidence is lacking [111,112].

Confirmation bias further compounds these effects by particularly driving professionals to selectively interpret information that aligns with their pre-existing beliefs and dismiss contradictory evidence, thereby negatively interfering with clinical reasoning [109,113,114]. These cognitive distortions not only bias clinical decision-making but also highlight the importance of fostering critical thinking and promoting evidence-based practices to mitigate the impact of these biases in musculoskeletal care.

Additional psychological effects relevant to the interpretation of CAMs include the Barnum effect and the Hawthorne effect. The Barnum effect, often exploited in pseudoscientific fields, occurs when individuals perceive vague and generic descriptions as highly accurate and personalized [115]. In practices like osteopathy and chiropractic, this effect leverages patients’ desires for diagnostic certainty and the need to identify predictable patterns in their health, fostering a sense of personal connection with the treatment and practitioner [116,117,118]. This perceived personalization can bypass critical reasoning, leading patients to trust vague explanations and interpret ambiguity as hidden meaning, which reinforces their belief in the therapy’s effectiveness despite a lack of scientific evidence [119]. As a result, emotional shortcuts in decision-making may drive continued adherence to such treatments, amplifying their perceived benefits [120].

The Hawthorne effect suggests that patients may modify their behavior and report therapeutic improvements simply due to their participation in treatment that includes regular and personalized follow-up by the therapist. This phenomenon is particularly evident in therapies where the evaluation criteria are subjective, such as pain or “dysfunction” [121]. Patients might exaggerate symptom improvements to align with the therapist’s expectations, leading to an overestimation of treatment efficacy in both effective and ineffective approaches [122,123]. Additionally, the exposure to a therapeutic ritual with promised results can shape symptom perception and evaluation, with many patients confirming expected changes to satisfy the therapist [123].

While these psychological factors are common to varying degrees among all individuals, certain traits appear to increase susceptibility to persuasion by these types of therapies and narratives. Characteristics such as agreeableness, introversion, and lack of premeditation are associated with greater susceptibility [124,125].

The studies used in this section employ diverse methodologies, including experimental research, surveys, qualitative studies, systematic reviews, and meta-analyses. They examine psychological elements such as placebo effects, cognitive biases, pseudoscientific beliefs, causal illusions, and the “Guru Effect”, with populations including CAMs users, patients with cancer or rheumatoid arthritis, university students, and physicians. Outcomes emphasize the cognitive, social, and cultural influences on beliefs in CAMs, highlighting mechanisms like prior expectations, causal illusions, and health-related judgments. However, limitations include small sample sizes, reliance on self-reports, and theoretical models alongside limited generalizability and potential biases such as recall or selection. The reader can find the studies analyzed in Appendix C.

### 5.4. The Effects Mediated by Context in Osteopathic and Chiropractic Practices for Musculoskeletal Care

Contextual factors in the therapeutic interaction between patients and healthcare providers encompass a wide range of elements. These factors extend beyond the specific actions of the treatment and include the physical environment, the quality of the relationship with the professional, the patient’s expectations, and the perceived credibility of the therapist. Additionally, rituals, healing cues, and other symbolic elements associated with the treatment process may also play a significant role in the patient’s response [126].

Often, expectations and perceptions of outcomes differ between the healthcare provider and the patient, which can lead to dissatisfaction with conventional care. This dissatisfaction is a key factor driving patients toward CAMs [127]. A patient’s expectations regarding the effectiveness of a therapy can significantly influence their perception of treatment outcomes [9,125]. If a person anticipates improvement, they are more likely to perceive a benefit, even if the treatment is objectively ineffective. Conversely, negative expectations can lead to unfavorable perceptions of outcomes, regardless of the treatment’s objective efficacy. Previous positive experiences can also enhance the response to treatment; for example, individuals who have had success with osteopathic or chiropractic manipulations are more likely to respond favorably to similar treatments in the future [128].

This influence of expectations has been observed by Bialosky [129] inducing positive and negative expectations in a group of patients with low back pain. Subjects who were given positive expectations about the effects of lumbar manipulation experienced a reduction in pain (hypoalgesia) in the treated area. Conversely, those who received negative expectation instructions reported an increase in pain perception (hyperalgesia) in the same region. Interestingly, these changes in pain perception were localized to the region where manipulation was expected to have an effect, with no significant impact on the lower extremities where no expectations were set.

**Figure 3:**
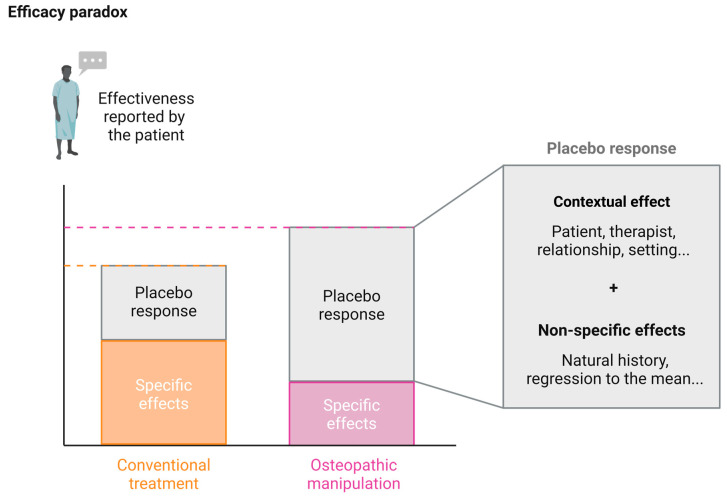
Efficacy paradox: The efficacy paradox focuses on the discrepancies between the results perceived by the patient and the real effectiveness of the intervention. In the case of osteopathy and chiropractic, it focuses on the interaction between the demonstrated specific effect of the technique and the significant influence of the placebo responses, mediated by the effects of non-specific factors and the therapeutic context, such as the complexity of the therapeutic ritual or the expectations generated by the therapist [46,132] Created in BioRender. F, M. (2025) https://BioRender.com/g83d974, accessed on 26 January 2025.

Rituals and ceremonies associated with CAMs can also influence the patient’s perception of treatment effectiveness, contributing to perceived therapeutic effects [116,130]. The “efficacy paradox” illustrates how a complex intervention, such as visceral manipulation or craniosacral mobilization, may have minimal specific effects but a large placebo effect. In contrast, conventional treatment with moderate specific effects but a smaller placebo effect may be perceived as less effective by the patient [130]. The complexity of an intervention often correlates with its perceived effectiveness (Figure 3).

This efficacy paradox has been studied in recent studies. Some patients report significant pain relief after osteopathy and chiropractic sessions primarily based on manipulation. These positive outcomes are short-term and largely attributed to placebo response, mainly driven by personalized care, the therapist–patient relationship, the time dedicated by the practitioner, and the perception of specificity for a particular issue. However, current evidence indicates no significant differences in their application methods, and their use should be prioritized based on patient comfort and preferences [131]. In contrast, therapeutic exercise has strong evidence supporting its effectiveness in improving pain and function in the long term. However, its effects may take longer to manifest and require active patient commitment, which can reduce adherence and the perceived outcomes.

Thus, the positive outcomes perceived by some individuals during the application of certain low-value techniques appear to be more related to the inherent characteristics of the therapeutic process than to the physical intervention itself. These characteristics may include the environment in which the therapy is conducted, the theoretical framework underlying the approach, the evaluation performed in each session, therapeutic touch, and the patient’s active involvement in their treatment [133,134,135]. Therefore, it is crucial to assess the contextual effects that may influence the individual, their condition, the intervention, and the outcome to determine the relevance of a particular intervention in each specific clinical setting [136]. These factors are often present in practices such as osteopathy and chiropractic, and they can lead to confusion on the part of the therapist regarding the actual effects of the applied techniques [95]. While the perceived benefits may be substantial, it is important for practitioners to distinguish between the therapeutic value of contextual factors and the specific efficacy of the techniques themselves.

### 5.5. Neurobiological Basis of Contextual Effects in Osteopathic and Chiropractic Practices for Musculoskeletal Care

The effects mediated by the context, such as placebo and nocebo effects, are modulated by various neurobiological systems, including opioid, endocannabinoid, and dopaminergic systems, which play an essential role in the modulation of pain and reward pathways [79,137,138]. These systems contribute to the analgesic effects observed in response to treatments, even in the absence of a specific intervention. Furthermore, placebos can influence serotonergic pathways, affecting emotional regulation and mood [137,139]. Placebo effects are also mediated by the interaction between cholecystokinin (CCK) and endogenous opioids, which regulate both positive and negative effects [138,140].

The neural modulation arising from these contextual effects involves a functional connection between several brain regions, such as the dorsolateral prefrontal cortex (CPFDL), the rostral anterior cingulate cortex (rACC), and subcortical regions such as the hypothalamus (HYP), amygdala (AMYG), and periaqueductal gray (PAG), which play a crucial role in placebo-induced analgesia [79,141,142]. During placebo analgesia, decreased neural activity is also observed in pain-associated regions, such as the thalamus and primary somatosensory cortex, and could potentially contribute to a subjective reduction in pain perception [78]. Furthermore, placebo effects also modulate spinal cord activity and descending analgesia pathways, reinforcing the influence of contextual factors on pain relief [143,144]. There are studies suggesting that brain morphology and functional connectivity can predict individual responses to placebo-induced analgesia [145,146]. Conversely, abnormal activation of the hippocampus (Hp) has been linked to nocebo effects, as this region, along with the AMYG, is involved in processing emotions such as anxiety and distress [79].

**Figure 4:**
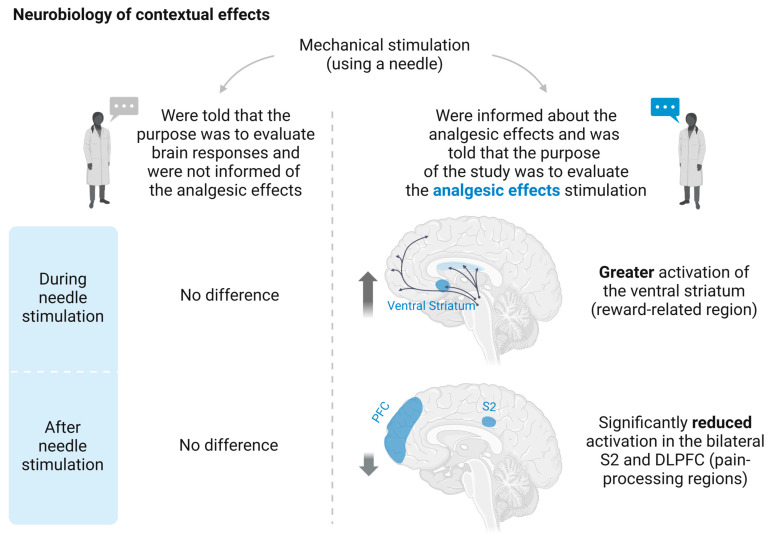
Neurobiology of Contextual Effects: The instructions given to patients and the expectations created about a health intervention seem to trigger neurobiological changes that align with the patient’s perception of the treatment’s effectiveness. This phenomenon highlights the significant role of cognitive and contextual factors in shaping therapeutic outcomes. Studies suggest that patient expectations can influence both neurophysiological responses and perceived benefits [147]. Created in BioRender. F, M. (2025) https://BioRender.com/g83d974, accessed on 26 January 2025.

Differing instructions and expectations regarding the same procedure can alter the nervous system’s response. It has been observed that when people are informed about the therapeutic efficacy of a technique, there is an increase in activity in regions such as the ventral striatum, associated with the reward circuit, and a decrease in activity in secondary somatosensory regions and the right dorsolateral prefrontal cortex. However, this change in activity does not occur when people are told that the technique may be painful or ineffective [147] (Figure 4). These findings suggest that the therapeutic context may have a more significant impact than the intervention itself.

While this evidence marks significant progress in understanding these therapeutic phenomena, research findings remain heterogeneous, and further exploration is needed to better understand the neurobiological mechanisms of these effects [148].

For the analysis of Section 5, the following articles were analyzed, spanning various study types, including narrative reviews, randomized controlled trials, systematic reviews, pilot studies, and meta-epidemiological analyses. They examine placebo and contextual effects in CAMs, with a focus on interventions like osteopathy, chiropractic and treatments for chronic pain and migraines. Populations include patients with chronic and musculoskeletal pain as well as general recipients of CAMs. Key outcomes highlight the role of placebo, nocebo, and contextual factors in shaping treatment perceptions and outcomes, particularly discrepancies in patient expectations. Common limitations include reliance on theoretical frameworks, small sample sizes, lack of control groups, and high heterogeneity in study designs, reducing applicability to specific populations. The reader can find the studies analyzed in Appendix C, Appendix D and Appendix E.

## 6. Ethical Considerations of the Effects Mediated by Context and Placebo as First-Line Musculoskeletal Therapy

The potential mechanisms underlying the effects of osteopathy and chiropractic in musculoskeletal care remain a matter of debate. On one hand, some studies suggest that specific biological mechanisms may play a role in these therapies, but these mechanisms are poorly understood and require further research [28,76]. On the other hand, most recent evidence highlights the biological implausibility of these CAMs. Even practitioners in these fields exposed the challenges in designing convincing and contextually relevant control interventions, which can significantly influence placebo effects across study groups [132]. Consequently, research findings must be interpreted with caution due to various methodological factors that contribute to a high risk of bias.

Integrating biologically implausible therapies, whose potential mechanisms may rely primarily on placebo effects (contextual and non-specific factors) into healthcare systems raises significant ethical dilemmas. CAMs such as osteopathy and chiropractic may lead to: (1) direct harms (i.e., side effects from cervical manipulation at maximum range of motion [69] or indirect harms (i.e., delaying an effective treatment); (2) emotional or financial harms, particularly for individuals with lower socioeconomic status; (3) wasted clinical resources; and (4) inequities in healthcare delivery.

Although osteopathy and chiropractic have built a substantial body of evidence over the years, clinical trials have consistently failed to demonstrate specific effects for improving various clinical conditions according to the most recent systematic reviews and meta-analyses [29,57,67,149]. For this reason, these therapies are still considered CAMs and have not evolved to conventional medicine. Consequently, their use by health care professionals for musculoskeletal care cannot be recommended. Scientific disciplines must rigorously challenge their own hypotheses and evolve their practices based on evidence. This case should not be an exception.

## 7. Implication for Clinical Practice

In many countries, these interventions are mandated to be performed by healthcare professionals, inadvertently lending them credibility through association with established musculoskeletal care practices. While it is unlikely that such therapies will disappear, nor is it the intention of this publication to advocate for their elimination, they must be critically evaluated to ensure their safety and efficacy. These practices should not be endorsed or integrated into musculoskeletal care without sufficient scientific validation.

At their current stage of development, most recent reviews and meta-analyses have shown CAMs such as osteopathy and chiropractic to be no better than placebo interventions or natural disease progression. Consequently, their clinical effectiveness is often overestimated, and they cannot be recommended as first-line treatments. This underscores the urgent need for robust, high-quality research to validate their efficacy and mechanisms. Rather than perpetuating a divide between disciplines, fostering constructive dialogue and collaboration between professionals is crucial for advancing integrated healthcare practices. This balanced approach will help ensure that only therapies with demonstrated value for musculoskeletal care are incorporated into mainstream practice, ultimately improving patient outcomes and promoting evidence-based decision-making.

### Limitations

This review, while comprehensive, has several limitations that merit acknowledgment. First, as a comprehensive review, the methodology allows for the inclusion of subjective interpretations during the synthesis of evidence. Despite efforts to minimize bias, the absence of a systematic review framework may reduce replicability and introduce selection bias into the literature analyzed.

Second, many of the studies reviewed exhibit significant methodological shortcomings. These include small sample sizes, inadequate control groups, and a reliance on observational designs rather than experimental approaches. Together, these factors weaken the robustness of the conclusions and hinder the generalization of findings to broader clinical contexts. The substantial heterogeneity in study designs and varying levels of methodological quality further exacerbate these challenges.

Third, although the focus of this review is the use of CAMs in musculoskeletal care—particularly osteopathy and chiropractic—some conjectures are derived from studies investigating different techniques or clinical scenarios. These findings may not always be directly transferable to the specific domain of interest, thereby limiting their applicability.

Fourth, certain articles reviewed on biological and psychological mechanisms are not specifically oriented toward CAMs, reducing their relevance and generalizability to this field.

Five, it is likely that the changes observed by osteopaths and chiropractors in their patients’ symptoms are due to unconscious long-term effects, such as placebo responses and non-specific factors, rather than specific effects of the interventions. These effects have not been studied in the long term, and there is no known evidence on the medium- and long-term impacts of these therapies.

Finally, the limited and methodologically diverse body of literature addressing the biological plausibility and potential mechanisms of osteopathy and chiropractic in musculoskeletal care has created theoretical gaps. These gaps necessitated reliance on broader or indirectly related studies to propose hypotheses, which may introduce additional uncertainty.

These limitations highlight the urgent need for future research employing rigorous and standardized methodologies. Well-designed randomized controlled trials, larger and more representative sample sizes, and consistent reporting standards are essential to address the gaps identified in the existing literature. Such efforts will contribute to a clearer understanding of CAMs’ role in musculoskeletal care and ensure that clinical practice remains grounded in scientifically validated principles.

## 8. Conclusions and Future Perspectives

The growth of musculoskeletal care as a clinical field reveals a dark side, or perhaps a gray area, characterized by the increasing adoption of therapies lacking robust scientific evidence, which undermines the legitimacy of the discipline. Scientific knowledge is essential for advancing healthcare; however, it is inherently limited. The absence of evidence does not equate to falsity, and what is accepted as valid today may be refuted tomorrow. Thus, it is crucial to distinguish between the veracity of a claim and the quality of the available evidence. Nevertheless, reliance on methodologies that lack scientific rigor, even when they occasionally yield promising results, is neither prudent nor ethical.

In many countries, osteopathy and chiropractic care are delivered by healthcare professionals, a fact that—combined with their association with validated musculoskeletal care practices—inadvertently lends these therapies credibility through authority bias. However, these interventions often rely on theoretical models and mechanisms of action rooted in questionable biological plausibility. Diagnoses such as “somatic dysfunctions” or “vertebral subluxations” are inconsistent with established scientific knowledge. Conducting research based on these concepts poses significant challenges to validating the treatments proposed by these therapies, especially when they are founded on implausible anatomical and physiological beliefs.

Studies evaluating the clinical effects of these interventions are frequently marred by significant biases, including small sample sizes, the absence of standardized control groups, the use of inadequate assessment tools, and a reliance on observational designs. Furthermore, the lack of proper planning for controls or placebos can lead to erroneous interpretations by introducing contextual and non-specific factors that alter the dynamics between participants and therapists during research.

Key contextual and non-specific factors influencing outcomes include participants’ expectations, amplified therapeutic rituals in the research setting, prior experiences with similar interventions, interactions with the research team, and variables such as the number, type, and timing of manual interventions. Additional influences include placebo effects, regression to the mean, and the natural history of the condition. These limitations hinder the generalizability of findings and reduce confidence in conclusions regarding the clinical effectiveness of these interventions.

Based on the current body of evidence, neither osteopathy nor chiropractic care can be recommended as first-line treatments for musculoskeletal conditions. Nonetheless, these practices are likely to remain part of clinical care due to cultural, historical, political, and patient preference factors. Therefore, it is imperative that these therapies undergo critical evaluation and are integrated into healthcare only when their safety and clinical efficacy are supported by rigorous scientific validation. By doing so, healthcare professionals can uphold their legitimacy, improve therapeutic outcomes, and foster informed decision-making.

Future research should prioritize addressing the limitations of the existing evidence base. This involves designing well-structured randomized controlled trials with larger, representative sample sizes and adequately planned control groups. Additionally, studies should incorporate approaches that consider contextual and non-specific factors, which, despite being often overlooked, appear to significantly impact clinical outcomes. Understanding these factors will contribute to a more accurate interpretation of the specific effects of osteopathic and chiropractic interventions. Furthermore, exploring advanced technologies—such as neurophysiological data analysis and its correlation with clinical outcomes—can provide greater precision in understanding the mechanisms of action of these therapies. Longitudinal studies are also essential to assess the long-term effects of these interventions, especially when integrated with evaluations of contextual and non-specific influences, which have been underexplored to date.

Finally, implementing evidence-based practices should be a priority, requiring healthcare professionals to actively stay informed and critically assess the methodological quality of published studies. To this end, we advocate for fostering a spirit of transdisciplinary and interdisciplinary collaboration among healthcare professionals and researchers to enhance methodological approaches and generate new evidence that optimizes clinical practice. Through this collective effort, the field of musculoskeletal healthcare can advance, promoting patient safety and delivering more effective treatments grounded in the best available scientific evidence.

## Appendix A. Biological Plausibility of CAMs

**Author (Year)**	**Study Type**	**CAMs**	**Population**	**Outcomes and Limitations**
Crow WT et al. (2009) [31]	Observational study	Osteopathy	Human calvarial structures	Outcomes:
				Statistically significant changes in cranial area.
				Changes above the resolution threshold of the MRI scanner (0.898 mm/pixel).
				Limitations:
				Limited resolution of the MRI scanner used.
				Small sample size (20 participants).
Mirtz et al. (2009) [42]	Epidemiological Review	Chiropractic	General chiropractic	Outcomes:
				Evaluated chiropractic subluxation using Hill’s causation criteria, concluding it lacks validity as a disease cause.
				Limitations:
				Based on theoretical application without experimental data; potentially subjective interpretations.
Homola (2013) [40]	Commentary/Review	Chiropractic	General chiropractic	Outcomes:
				Critiqued the concept of “vertebral manipulation” in chiropractic, pointing out lack of scientific evidence for “vertebral subluxation”.
				Limitations:
				No original research data provided.
				Findings are interpretive and based on existing literature, introducing potential bias.
Homola (2016) [41]	Narrative review	Pediatric chiropractic	Pediatric population	Outcomes:
				Criticized “vertebral subluxation” concept in pediatric chiropractic care, emphasizing lack of scientific support.
				Discussed potential health risks for children and need for appropriate medical referral.
				Limitations:
				No original research data provided.
				Findings are interpretive and based on existing literature, introducing potential bias.
Horton (2015) [33]	Clinical review	Visceral Mobilization therapy (VMT)	General population, focused on pelvic dysfunctions	Outcomes:
				Identified potential clinical applications of VMT in treating genitourinary dysfunction.
				Outlined some clinical evidence supporting VMT for genitourinary and pelvic dysfunction.
				Limitations:
				Evidence limited primarily to case reports and observational studies.
				Lacks robust clinical and experimental trials.
				Effectiveness for specific conditions remains speculative.
				Proposed biological mechanisms lack empirical support.
Côté P et al. (2020) [38]	Commentary/Review	Chiropractic	General population	Outcomes:
				Data linking chiropractic manipulation to immune system are unreliable.
				Lack of biological plausibility in relationship between chiropractic manipulation and immune system.
				Limitations:
				Does not provide new experimental data on biological mechanisms, relying on previous reviews and expert opinions.
Nim et al. (2021) [43]	Systematic review	Spinal manipulation	Patients with spinal pain	Outcomes:
				No significant difference between targeted and non-targeted manipulation sites, suggesting specificity may not impact treatment effectiveness.
				Limitations:
				Limited by small number of studies (10) and high variability in study designs.
				Differences in patient populations and protocols impact consistency of findings.
Requena-García J et al. (2021) [32]	Educational Model validation	Cranial osteopathy	Students learning cranial osteopathy	Outcomes:
				Relationship found between therapist experience and reliability in palpating cranial movements.
				Limitations:
				Use of cadaveric model limits transferability to real clinical situations.
				Variations in cranial movement, measured in microns, probably not perceptible by therapists.
Consorti G et al. (2023) [37]	Conceptual/Theoretical study	Somatic dysfunction	General population	Outcomes:
				Presents an enactive theoretical framework on “osteopathic dysfunction”.
				Limitations:
				Lacks evidence on proposed potential neurobiological mechanisms.
				Does not establish clear relationship between proposed mechanisms and clinical situation.
Bordoni B, Escher AR. (2023) [30]	Review	Cranial osteopathy	General population	Outcomes:
				Inconsistencies in PRM theory highlighted.
				Cerebrospinal fluid (CSF) movement is inhomogeneous both centrally and peripherally.
				Limitations:
				No evidence that CSF movement is detectable by palpation.
				Strong suspicion that spheno-occipital synchondrosis is incapable of moving sacrum.
Hidalgo D et al. (2024) [35]	Narrative review	Osteopathy	Not applicable	Outcomes:
				Examines concept of “anatomical possibilism” in osteopathy.
				Argues this approach may lead to unsupported diagnostic and treatment practices.
				Emphasizes need for osteopathic interventions based on rigorous scientific evidence.
				Limitations:
				Lacks empirical data and relies on theoretical critique, which may introduce interpretative bias.

## Appendix B. Clinical Effectiveness of Osteopathy and Chiropractic in Musculoskeletal Care

**Author (Year)**	**Study Type**	**Musculoskeletal Care Practice**	**Population**	**Outcomes and Limitations**
Ernst (2008) [68]	Evaluation of chiropractic practices, focusing on spinal manipulation and subluxation concepts	Chiropractic care	General population	Outcomes:
				Chiropractic care, particularly spinal manipulation, has been associated with frequent mild adverse effects and rare severe complications.
				Subluxation and spinal manipulation lack scientific backing.
				Spinal manipulation has only shown effectiveness for back pain.
				Many chiropractors treat non-musculoskeletal conditions without proven efficacy.
				The therapeutic value of chiropractic remains unproven beyond reasonable doubt.
				Limitations:
				Lack of empirical evidence for effectiveness beyond back pain.
				Incidence of severe complications from spinal manipulation is unknown.
				The review relies on existing literature, lacking new empirical data.
				No evidence for cost-effectiveness of chiropractic care.
Gross et al. (2010) [63]	Systematic review	Cervical manipulation and mobilization for neck pain	Adults with neck pain	Outcomes:
				Moderate quality evidence showed that cervical manipulation and mobilization produced similar effects on pain, function, and patient satisfaction at intermediate-term follow-up.
				Low quality evidence suggested cervical manipulation provided greater short-term pain relief than control.
				Low quality evidence also supported thoracic manipulation for pain reduction and improved function in acute pain and chronic neck pain.
				Optimal technique and dose need to be determined.
				Limitations:
				Low quality evidence for some outcomes.
				Methodological quality of studies varied (33% had low risk of bias).
				Limited evidence on the optimal technique and dose for manipulation and mobilization.
Walker et al. (2010) [64]	Systematic review	Combined chiropractic interventions	Adults with chronic low back pain	Outcomes:
				Chiropractic interventions improved short- and medium-term pain and disability in acute and subacute LBP compared to other therapies.
				No significant difference for long-term pain or disability.
				Small improvements in short-term disability with chiropractic interventions compared to other therapies.
				No difference for chronic LBP.
				Limitations:
				Studies with high risk of bias.
				Small improvements in outcomes, with no clinically meaningful difference compared to other treatments.
				Limited evidence for chronic LBP and mixed populations.
				Need for better quality trials.
Rubinstein et al. (2011) [150]	Systematic review and meta-analysis	Spinal manipulative therapy (SMT) for chronic low back pain	Adults with chronic low back pain	Outcomes:
				High-quality evidence suggests SMT has a small, statistically significant but not clinically relevant effect on pain relief and functional status in the short term compared to other interventions.
				Varying evidence for the effectiveness of SMT when added to other interventions.
				Very low-quality evidence for SMT’s efficacy compared to inert or sham SMT.
				Limitations:
				No evidence of serious complications, but limited data on recovery, return-to-work, quality of life, and costs of care.
				Inconsistent quality of evidence for various outcomes.
				Sparse data on long-term effects and overall cost-effectiveness.
				High heterogeneity and variability in the studies.
Ernst (2012) [67]	Review	Chiropractic spinal manipulation	Patients with musculoskeletal and non-musculoskeletal conditions.	Outcomes:
				Cautiously positive evidence for chiropractic spinal manipulation in treating low back pain and neck pain.
				Negative results for non-spinal conditions such as asthma and dysmenorrhoea.
				Cochrane reviews generally considered reliable but show limited evidence for effectiveness in certain conditions.
				Limitations:
				Clinical and statistical heterogeneity across studies prevented meta-analysis.
				Limited evidence for non-spinal conditions.
				Heterogeneity in the included studies made it difficult to draw definitive conclusions.
Ernst (2012) [52]	Systematic review	Craniosacral therapy (CST)	Various disorders	Outcomes: The review found that CST showed no substantial evidence of effectiveness for any condition. While low-quality studies suggested potential positive effects, the high-quality trial did not demonstrate any significant benefits. Limitations: The review included six studies, most of which had a high risk of bias. The methodological quality was generally poor, with only one study of higher quality. The positive effects suggested by low-quality studies were not corroborated by higher-quality trials, leading to doubts about the validity of CST’s clinical benefits.
Ajimsha et al. (2013) [59]	Randomized controlled trial	Myofascial release	Nursing professionals with chronic lower back pain	Outcomes:
				MFR showed greater improvement in pain and disability compared to the control group.
				MFR group had a 53.3% reduction in pain and 29.7% reduction in disability at week 8, with continued improvement at week 12.
				73% of MFR group had ≥50% pain reduction.
				Limitations:
				Single-blind design could introduce bias.
				No placebo for control group, only sham MFR.
				Small sample size (80 participants).
				The study did not address long-term effects beyond 12 weeks.
				The control group received sham MFR, which may not fully mimic the standard care.
Franke et al. (2014) [47]	Systematic review and meta-analysis	Osteopathic manipulative treatment (OMT)	Adults with non-specific low back pain	Outcomes: Moderate-quality evidence showed OMT significantly improved pain and functional status in acute and chronic nonspecific LBP. Limitations: Low evidence quality limits generalizability. The small number of included trials limits robustness. Future research requires larger, high-quality randomized controlled trials (RCTs) with robust control groups.
Guillaud et al. (2016) [53]	Systematic review	Craniosacral therapy (CST)	Various disorders	Outcomes:
				Diagnostic procedures used in cranial osteopathy are unreliable in many cases.
				For efficacy, the review found that the studies had significant methodological flaws, with only three studies showing low risk of bias.
				These studies failed to rule out non-specific effects, and no strong evidence supported the efficacy of cranial osteopathy.
				Limitations:
				Diagnostic reliability, there was inconsistency in the results, indicating a lack of reliability in cranial osteopathy diagnostics. The methodological quality of the included studies was generally low.
				High risk of bias.
				Low quality of the studies.
				The heterogeneity in study designs and methodologies may limit the generalizability of the findings.
Arguisuelas et al. (2017) [60]	Randomized controlled trial	Myofascial release	Adults with nonspecific chronic low back pain	Outcomes:
				Significant improvements in pain (SF-MPQ) and sensory subscale, compared to sham MFR.
				Disability and fear-avoidance beliefs significantly decreased in the MFR group compared to the control.
				No differences in VAS scores between groups.
				Limitations:
				The clinical relevance of the improvements is uncertain due to the 95% CI overlapping the minimal clinically important differences.
				The study was limited to a small sample size (54 participants).
				Short duration of the intervention (4 sessions).
				Lack of long-term follow-up data.
Kranenburg HA et al. (2018) [69]	Systematic review	Cervical spine manipulation (CSM) and mobilization	Patients with neck pain and headache	Outcomes:
				Identified characteristics of patients, practitioners, treatment process, and adverse events (AE).
				Cervical arterial dissection (CAD) reported in 57% of cases, with women more at risk.
				Limitations:
				Poor description of patient characteristics and under-reporting of cases.
				Further research needed for uniform AE registration using standardized terminology.
Rubinstein SM et al. (2019) [65]	Systematic review and meta-analysis	Spinal manipulative therapy (SMT)	Patients with chronic low back pain	Outcomes:
				SMT produces similar effects to recommended therapies for short-term pain relief and moderate improvement in function.
				Compared to non-recommended therapies, SMT shows a small to moderate improvement in function but minimal pain relief.
				Evidence for sham SMT is of low quality, suggesting uncertain effects.
				Musculoskeletal adverse events were transient and mild to moderate in severity.
				Limitations:
				High heterogeneity between studies made it difficult to interpret some findings.
				Evidence for the effectiveness of sham SMT was of very low quality.
				Most studies did not systematically report adverse events.
				Some results were not clinically relevant despite statistical significance. comparisons, heterogeneity in comparison treatments.
Rehman et al. (2020) [48]	Systematic review and meta-analysis	Osteopathic manual therapy (OMT)	Patients with chronic pain	Outcomes:
				Some improvement in pain and functional outcomes findings limited by inconsistent methodologies.
				OMT demonstrated no significant impact compared to physiotherapy or gabapentin for any measured outcomes.
				Limitations:
				Small sample sizes.
				Variability in techniques and outcomes.
				Heterogeneity among comparator treatments and outcome measures reduces generalizability.
Farra et al. (2021) [49]	Systematic review and meta-analysis	Osteopathic interventions (OMT, MFR, CST, OVM)	Patients with chronic non-specific low back pain	Outcomes:
				Osteopathic interventions are more effective than control treatments in reducing pain and improving functional status.
				Myofascial release (MFR) showed the most effective results for pain reduction, with moderate-quality evidence.
				Osteopathic manipulative treatment (OMT) showed a low-quality effect in pain reduction.
				Craniosacral therapy (CST) and osteopathic visceral manipulation (OVM) showed limited evidence for efficacy.
				Limitations:
				None of the studies were judged at low risk of bias (RoB).
				Low to very-low-quality evidence for some treatments, particularly for OMT and CST.
				Limited diversity in osteopathic treatment types, which hinders generalization of findings.
				Further high-quality trials are needed to better compare different osteopathic techniques.
Nguyen et al. (2021) [61]	Randomized clinical trial	Osteopathic manipulative treatment (OMT)	Adults with nonspecific subacute or chronic low back pain (LBP)	Outcomes:
				The standard OMT group showed a mean reduction in LBP-specific activity limitations of −4.7 points (Quebec Back Pain Disability Index) at 3 months, significantly better than sham OMT group (−1.3 points).
				No significant difference in pain reduction at 3 and 12 months.
				Serious adverse events reported in both groups but not related to OMT.
				Limitations:
				The effect of OMT on LBP-specific activity limitations is small and its clinical relevance is questionable.
				No significant differences found for secondary outcomes such as pain and quality of life.
				The study lacks long-term efficacy data, and the sham OMT may not fully replicate standard OMT in terms of patient expectations.
Farra et al. (2022) [50]	Systematic review and meta-analysis	Osteopathic manipulative treatment (OMT)	Adults with non-specific neck pain	Outcomes:
				Osteopathic interventions showed statistically significant improvements in pain levels and functional status compared to no intervention or sham treatments.
				Limitations:
				Small sample sizes.
				Difficulty standardizing techniques.
				Evidence quality was rated as “very low.”
Lotfi et al. (2023) [51]	Literature review	Osteopathic manipulative teatment (OMT)	Patients with irritable bowel syndrome (IBS)	Outcomes:
				The review suggested that OMT may reduce IBS symptoms such as abdominal pain, bloating, and irregular bowel movements. Improvements were attributed to potential modulation of visceral function and nervous system responses.
				Limitations:
				Evidence relied on a small number of studies with varying methodologies and quality.
				Lack of high-quality RCTs and limited generalizability.
				The findings were based on limited and mixed evidence.
Ceballos-Laita et al. (2023) [58]	Systematic review and meta-analysis	Visceral osteopathy	Adults with low back pain	Outcomes:
				Visceral osteopathy did not show significant improvements in pain intensity, disability or physical function.
				High heterogeneity found in the pain intensity outcome.
				Limitations:
				High risk of bias in the included studies.
				Lack of high-quality studies evaluating the effectiveness of visceral osteopathy for LBP.
				The small number of studies included (5 studies, 268 patients) and heterogeneity in outcomes limit the reliability of conclusions.
Buffone et al. (2023) [55]	Systematic review and meta-analysis	Osteopathic manipulative treatment (OMT)	Irritable bowel syndrome (IBS)	Outcomes:
				OMT showed statistically significant improvement in abdominal pain and constipation, with effect sizes.
				OMT was not superior to control for other IBS symptoms such as severity of IBS, Likert scale ratings, and diarrhea.
				The quality of evidence was deemed “low” for abdominal pain and constipation, and “very low” for diarrhea.
				The evidence did not support the superiority of OMT for all IBS symptoms,
				OMT was found to be safe with no major adverse effects.
				Limitations:
				The methodological quality of the included studies was generally low.
				High risk of bias.
				Low quality of the studies.
				The heterogeneity in study designs and methodologies may limit the generalizability of the findings.
Silva et al. (2023) [54]	Systematic review	Visceral fascial therapy	Patients with visceral dysfunctions	Outcomes:
				Visceral Fascial Therapy showed effectiveness in reducing pain in patients with low back pain when combined with standard physical therapy, and in reducing gastroesophageal reflux symptoms in the short term.
				Limitations:
				High risk of bias.
				Low quality of the studies.
				The heterogeneity in study designs and methodologies may limit the generalizability of the findings.
				The evidence for the effectiveness of Fascial Therapy targeting visceral dysfunctions remains insufficient to support widespread clinical use.
Ceballos-Laita et al. (2024) [56]	Systematic review and meta-analysis	Craniosacral therapy	Various disorders	Outcomes:
				CST produced no statistically significant or clinically relevant changes in pain or disability for musculoskeletal disorders like headache, neck pain, low back pain, pelvic girdle pain, and fibromyalgia.
				CST was also ineffective for non-musculoskeletal disorders like infant colic, cerebral palsy, and visual function deficits.
				Limitations:
				While the literature searches were thorough, it is impossible to ensure no relevant studies were missed.
				The inclusion of a wide range of diverse conditions complicates the interpretation of the results and weakens the strength of the conclusions.
				There was considerable heterogeneity across the included RCTs in terms of treatment duration and outcome variables, which may limit the validity of the quantitative syntheses.
Bonanno et al. (2024) [28]	Scoping review	Osteopathic manipulative treatment (OMT)	Healthy individuals and patients with chronic musculoskeletal pain	Outcomes:
				OMT appears to influence brain activity in healthy individuals and more significantly in patients with chronic musculoskeletal pain. The review includes studies involving fMRI, EEG, and brain connectivity analysis.
				Limitations:
				Limited number of included studies with mixed designs (RCTs, pilot studies, and crossover studies).
				Studies had variable methodologies and sample sizes.
				More high-quality RCTs are needed to confirm the findings on brain activity and neurophysiological effects of OMT.
Carrasco-Uribarren et al. (2024) [149]	Systematic review and meta-analysis	Craniosacral therapy	Patients with headache disorders	Outcomes:
				Craniosacral therapy resulted in a statistically significant but clinically unimportant change in pain intensity.
				No significant change in disability or headache effect.
				Very low certainty of evidence.
				Limitations:
				The evidence quality was downgraded to very low.
				Small number of studies (4 studies) with a limited sample size.
				Pain reduction was statistically significant but clinically irrelevant.
				No significant effects on disability or headache effect.
Farra et al. (2024) [76]	A comprehensive mapping review	Osteopathic manipulative treatment (OMT)	General population with various conditions	Outcomes:
				The review found biological effects induced by OMT, particularly neurophysiological and musculoskeletal changes.
				Limitations:
				Significant variability in study designs, participant conditions, OMT protocols, and documented biological effects.
				The diverse nature of the studies complicates the ability to draw definitive conclusions.
				The review suggests the need for further research to clarify whether these changes are specifically due to OMT and to corroborate their clinical implication.
Ceballos-Laita et al. (2024) [57]	Systematic review and meta-analysis	Visceral osteopathy	Patients with various musculoskeletal and non-musculoskeletal conditions	Outcomes:
				Visceral osteopathy showed no significant improvement in musculoskeletal conditions such as low back pain, neck pain, or urinary incontinence.
				No effect was found for non-musculoskeletal conditions like irritable bowel syndrome, breast cancer, or preterm infants.
				Studies had high risk of bias and low-to-very low certainty of evidence.
				Limitations:
				Most studies were at high risk of bias.
				Certainty of evidence was downgraded to low or very low.
				No statistically significant changes in outcomes.
				Positive results in non-musculoskeletal conditions were based on flawed studies.
				Many studies did not report adverse events.

## Appendix C. Effects and Potential Mechanisms of Osteopathy and Chiropractic in Musculoskeletal Pain: Placebo Response: Interaction Between Non-Specific and Contextual Factors in Osteopathic and Chiropractic Practices for Musculoskeletal Care

**Author (Year)**	**Study Type**	**Mechanism and Effect Placebo**	**Population**	**Outcomes and Limitations**
Benedetti et al. (2005) [140]	Narrative review	Neurobiological mechanisms of placebo effect	General population	Outcomes:
				Identified several neurobiological mechanisms underlying placebo effects.
				Highlighted the role of opioid and non-opioid neurotransmitter systems in placebo analgesia.
				Described how placebo effects can modulate various physiological systems beyond pain, including motor performance and immune responses.
				Limitations:
				The complexity of placebo mechanisms makes it challenging to isolate specific factors.
				Many studies cited were conducted in experimental settings, which may not fully reflect clinical realities.
				The review doesn’t address potential differences in placebo mechanisms across different medical conditions or populations.
Eippert (2009) [88]	Experimental	Descending modulation mechanisms in placebo analgesia	Healthy volunteers	Outcomes:
				Placebo analgesia is associated with increased activity in the dorsolateral prefrontal cortex, rostral anterior cingulate cortex, and periaqueductal gray matter.
				This activation was reversed by the opioid antagonist naloxone, indicating the involvement of endogenous opioids.
				The study provided indirect evidence that opioidergic descending pain control circuits underlie placebo analgesia.
				Limitations:
				Relatively small sample size.
				The study was conducted on healthy volunteers, which may limit generalizability to clinical pain conditions.
				The use of experimental pain may not fully reflect chronic pain experiences.
				The study focused on short-term effects and did not address long-term placebo responses.
Schweinhardt et al. (2009) [89]	Experimental	Placebo analgesia and personality traits	Healthy volunteers	Outcomes:
				The study suggests that the anatomy of the mesolimbic reward system may predispose individuals to placebo analgesia.
				Found a correlation between placebo analgesic responses and gray matter density in the mesolimbic reward system: ventral striatum, insula, and medial prefrontal cortex.
				Identified a link between placebo analgesia and personality traits: ego-resiliency and straightforwardness.
				Limitations:
				Small sample size limits generalizability.
				The study was conducted on healthy volunteers, which may not reflect responses in clinical pain populations.
				The correlational nature of the findings limits causal inferences.
				The study focused on brain structure rather than function, which may not capture the full complexity of placebo responses.
Hróbjartsson, Gøtzsche (2010) [91]	Systematic review and meta-analysis	Placebo interventions for all clinical conditions	Patients with various clinical conditions	Outcomes:
				Found no evidence that placebo interventions have important clinical effects in general.
				Possible small benefits in studies with continuous subjective outcomes and for the treatment of pain.
				In general, no significant effects outcomes.
				Limitations:
				High heterogeneity among studies.
				Difficulty in distinguishing genuine placebo effects from bias.
				Lack of data on harms of placebo interventions.
Stein et al. (2012) [146]	Experimental	White matter integrity and placebo analgesia	Healthy volunteers	Outcomes:
				Found that white matter integrity of the descending pain modulatory system, particularly in the dorsolateral prefrontal cortex and rostral anterior cingulate cortex, predicted individual differences in placebo analgesia.
				Suggests a neuroanatomical basis for variability in placebo responses.
				Limitations:
				Small sample size.
				Study conducted on healthy volunteers, limiting generalizability to clinical populations.
				Focus on acute experimental pain may not reflect chronic pain conditions.
Amanzio (2013) [142]	Meta-analysis	Brain connectivity in placebo analgesia	Healthy volunteers	Outcomes:
				Identified consistent activation patterns associated with placebo analgesia, including in the rCCA, CPFDL, and PAG.
				Deactivation was observed in areas processing pain.
				The study supports the involvement of opioid and non-opioid mechanisms in placebo analgesia.
				Limitations:
				Focus on experimental pain in healthy volunteers may limit generalizability to clinical pain.
				Heterogeneity in study designs and analysis methods across included studies.
				The meta-analysis was based on a relatively small number of neuroimaging studies
Atlas, Wager (2014) [141]	Meta-analysis	Placebo analgesia and expectancy-based pain modulation	Healthy volunteers	Outcomes:
				Consistent placebo-induced reductions in pain-related brain regions (dorsal anterior cingulate, thalamus, insula, amygdala, striatum)
				Increased activation in prefrontal cortex, midbrain, and rCCA.
				Suggests placebo effects impact both pain processing and emotion/value systems.
				Limitations:
				Variability in experimental designs across studies.
				Focus on contrasts rather than correlations with behavior.
				Limited ability to determine causal mechanisms.
Büchel (2014) [145]	Perspective/Review	Placebo hypoalgesia and predictive coding	N/A (Not applicable)	Outcomes:
				Proposes a hierarchical Bayesian framework based on predictive coding to explain placebo hypoalgesia.
				Suggests that placebo hypoalgesia results from combining top-down prior expectations with bottom-up sensory signals.
				Emphasizes the importance of both the mean and precision of predictions and sensory signals.
				Reframes the ascending and descending pain systems as a recurrent system implementing predictive coding.
				Limitations:
				Conceptual framework, not an empirical study.
				Focuses only on acute pain in healthy individuals.
				Precise neurobiological implementation of the model remains speculative.
Colloca (2014) [78]	Narrative review	Placebo and nocebo responses in pain management	General population	Outcomes:
				The paper synthesizes mechanisms behind placebo and nocebo effects, particularly in pain management, highlighting the role of cognitive, emotional, and contextual factors in modulating pain perception.
				Neurobiological pathways (e.g., endogenous opioids, dopamine) are explored.
				Limitations:
				The study is a synthesis, lacking direct empirical data.
				It heavily relies on secondary sources, which may introduce bias in interpretation.
				The generalizability of findings across diverse clinical scenarios remains uncertain.
Peciña, Zubieta (2014) [139]	Narrative review	Molecular mechanisms of placebo responses in humans	Patients with various clinical conditions	Outcomes:
				The study investigates the role of the μ-opioid receptor system in mediating placebo analgesia.
				It identifies specific neurobiological pathways, showing that placebo effects are influenced by the brain’s pain and reward modulation systems.
				The interaction between dopamine and opioid pathways is highlighted in placebo responses.
				Limitations:
				This is a review paper, so it is based on secondary data and may be biased.
				Further research is needed to explore these mechanisms in diverse clinical populations.
Wager, Atlas (2015) [94]	Review	Neuroscience of placebo effects, focusing on context, learning, and health	General population	Outcomes:
				The review explores neural mechanisms of placebo effects, highlighting the role of the prefrontal cortex, endogenous opioid and dopamine pathways, and the influence of learning and context on treatment outcomes.
				Limitations:
				Lacks new empirical data and focuses broadly on neuroscience, limiting its applicability to specific clinical contexts like musculoskeletal care.
				Further research is needed to validate these mechanisms in diverse settings.
Cerritelli (2016) [132]	Systematic Review	Placebo/sham therapy in osteopathy	Healthy population and population with different clinical conditions.	Outcomes:
				Evaluation of the application of placebo and sham therapies in osteopathic clinical trials.
				The lack of standardized methods and variability in sham approaches across studies are highlighted.
				High heterogeneity in the design of placebo controls, making clear conclusions on the effectiveness of sham therapies difficult.
				Limitations:
				High risk of bias in studies, particularly in allocation, blinding and selective reporting.
				Variation in sham therapy methodologies and insufficient reported information make it difficult to assess placebo effects in osteopathy.
				A quantitative analysis could not be performed due to these methodological limitations.
				The article highlights the need to develop standardized guidelines for placebo controls in manual medicine trials.
Testa, Rossettini (2016) [83]	Narrative review	Placebo and nocebo effects in physiotherapy	General population undergoing physiotherapy	Outcomes:
				The review examines the neurobiology of placebo and nocebo effects in physiotherapy.
				It highlights the role of contextual factors, such as the physiotherapist’s and patient’s characteristics, the therapist–patient relationship, and the healthcare environment.
				Contextual factors are identified as key modulators of clinical outcomes.
				Focus is placed on enhancing placebo effects and minimizing nocebo effects in physiotherapy treatments.
				Limitations:
				The review is a narrative synthesis, relying on existing literature without new empirical data.
				It centers on general placebo and nocebo concepts but lacks specific experimental evidence.
				The clinical applicability of the discussed effects in physiotherapy remains unvalidated through direct experimentation.
Ashar (2017) [92]	Narrative review	Placebo mechanisms and affective appraisal	Not specified	Outcomes:
				This review provides an overview of the placebo effect and its underlying brain mechanisms, particularly how appraisals of treatments influence outcomes.
				It identifies how placebo treatments, including those for pain, engage a core network of brain regions associated with self-evaluation, emotion, and reward processing, within the default mode network.
				The review emphasizes that placebo effects work by modifying how people evaluate their symptoms and future well-being.
				Limitations:
				The review does not introduce new empirical data or clinical trials.
				The generality of the findings, based on cognitive and neural appraisals, limits its direct applicability to specific clinical conditions or populations.
Beedie et al. (2018) [93]	Editorial	The role of placebo effects in CAM use in sports medicine and physiotherapy	Athletes and practitioners (elite and non-elite)	Outcomes:
				This review discusses the role of placebo and nocebo effects in complementary and alternative medicine (CAM) in sports medicine, emphasizing the complexity and variability of placebo effects.
				It presents placebo mechanisms like dopamine and opioid systems.
				Highlights challenges in using placebo effects to legitimize CAM, including variability, negative placebo effects (nocebo), and ethical concerns around deception.
				Suggests “headroom” mechanisms: the capacity to respond to placebos could indicate reserve capacity for legitimate treatments.
				Limitations:
				The review is based on existing literature and lacks original empirical data.
				Limited to placebo mechanisms, not addressing the full spectrum of CAM effects or evidence.
				Caveats in using placebo mechanisms for CAM are not fully explored, especially with regards to practical application in sports physiotherapy.
				Some recommendations may not be directly applicable across all CAM practices.
Blasini et al. (2018) [82]	Narrative review	The role of patient-practitioner relationships in placebo and nocebo phenomena	Pain patients (general clinical setting)	Outcomes:
				Identifies the biopsychosocial factors influencing placebo and nocebo effects in the patient-practitioner relationship.
				Emphasizes the role of expectancies and contextual factors (verbal suggestions, conditioning, and social observation) in shaping therapeutic outcomes.
				Found that macro (cultural, societal) and micro (individual psychobiological traits) factors influence expectancies.
				Empathy, friendliness, and competence of the practitioner enhance positive expectancies and placebo effects.
				Patient-practitioner caring and warm interactions improve the therapeutic experience, particularly for pain patients.
				Limitations:
				The review is based on existing literature without new empirical data.
				Focuses on theoretical models, lacking direct experimental evidence in the clinical setting.
				Subjective interpretations and lack of systematic analysis may reduce generalizability of findings across different clinical populations.
				The review does not provide concrete guidelines for integrating these findings into clinical practice.
Cai, He (2019) [137]	Narrative review	Placebo effects and molecular biological components involved	General clinical setting	Outcomes:
				Summarizes the history and characteristics of placebo effects.
				Identifies key molecular components involved in placebo effects, including the dopamine, opioid, serotonin, and endocannabinoid systems.
				Introduces the concept of placebome, aiming to understand the genetic and molecular basis of placebo effects.
				Discusses placebome studies and the need for no-treatment control (NTC) to identify genetic targets.
				Limitations:
				The placebome concept is still in its early stages.
				Lacks experimental data and new empirical findings.
				No clinical trials were included to test the molecular findings in real clinical settings.
				Emphasizes theoretical bioinformatics analysis rather than practical evidence in the clinical context.
				Need for NTC-controlled placebo studies to validate results and further explore the genetic targets related to placebo effects.
Anderson, Stebbins (2020) [80]	Narrative review	Determinants of placebo effects and responses	General clinical and research settings	Outcomes:
				Explores intrinsic factors influencing placebo responses, including patient expectations, previous experiences, neural systems under treatment, personality traits, and situational factors.
				Identifies clinician determinants, such as empathy, perceived expertise, clinical relationship quality, and belief in treatment efficacy.
				Analyzes extrinsic factors, such as study design, advertising, branding, and cultural influences, highlighting their combined impact on placebo effects.
				Limitations:
				Provides a theoretical framework without new empirical evidence.
				Focuses on general determinants of placebo effects rather than specific contexts, such as musculoskeletal care.
				Does not evaluate how identified factors quantitatively influence placebo responses in clinical practice or research.
Crawford et al. (2021) [144]	Experimental study	Brainstem mechanisms involved in placebo analgesia and nocebo hyperalgesia	Healthy volunteers	Outcomes:
				Found altered activity in key pain modulatory brainstem nuclei during placebo and nocebo responses.
				Identified distinct recruitment of the PAG-RVM pathway during greater placebo analgesia and nocebo hyperalgesia.
				Demonstrated differential activation of the parabrachial nucleus and overlapping activation in the substantia nigra and locus coeruleus for both effects.
				Suggests that the PAG-RVM pathway influences pain modulation at the level of the dorsal horn.
				Limitations:
				Small sample size (N = 25) limits generalizability of findings. Study focuses on acute experimental pain, reducing relevance to chronic pain scenarios.
				Deceptive conditioning may introduce variability in participants’ responses.
				Findings are correlational, limiting causal inference about brainstem circuitry and pain modulation.
Shi et al. (2021) [79]	Experimental study	Placebo and nocebo responses in acute lower back pain (ALBP)	Healthy volunteers	Outcomes:
				Significant differences in VAS pain scores observed for placebo and nocebo interventions compared to baseline and between placebo and nocebo groups.
				Placebo network involves negative lagged-temporal correlation between the DLPFC, secondary somatosensory cortex, ACC, and IC.
				Positive correlations were found between IC, thalamus, ACC, and SMA.
				Nocebo network includes positive correlations among primary somatosensory cortex, caudate, DLPFC, and SMA.
				Placebo response engages the reward system, inhibits the pain network, and activates opioid-mediated analgesia and emotion pathways.
				Nocebo response deactivates emotional control and primarily engages pain-related pathways.
				Verified that placebo and nocebo networks share brain regions but also have distinct features.
				Limitations:
				Small sample size (N = 20) limits the generalizability of findings.
				Study was conducted in healthy individuals, which may not reflect responses in clinical populations with chronic pain.
				Correlational nature of findings limits causal interpretations.
				fMRI-based GCA may be influenced by methodological biases, such as signal variability and lag-time estimation.
Thomson et al. (2021) [95]	Editorial/review	Exploration of contextual factors (CFs) in osteopathy and musculoskeletal care	N/A	Outcomes:
				Highlights the critical role of contextual factors such as clinician habits, patient expectations, therapeutic relationships, and treatment environments in shaping clinical outcomes.
				Suggests CFs influence outcomes via placebo and nocebo effects.
				Discusses the lack of CF awareness in osteopathic education and its implications for enhancing patient outcomes.
				Proposes research directions for better integration and evaluation of CFs in osteopathy and healthcare.
				Limitations:
				The study is narrative and does not include new empirical data or quantitative analysis.
				Limited generalizability due to its focus on osteopathy, though findings may apply broadly.
				Recommendations are theoretical and require further research validation through robust empirical methods.
				Does not specify direct evidence linking CF manipulation to improved outcomes in osteopathy.
Zunhammer et al. (2021) [148]	Systematic meta-analysis	Neural systems and brain mechanisms underlying placebo analgesia, based on experimental fMRI studies	Healthy volunteers	Outcomes:
				Identifies placebo analgesia as a multifaceted phenomenon involving multiple brain areas, including ventral attention networks (mid-insula), somatomotor networks (posterior insula), thalamus, habenula, mid-cingulate cortex, and supplementary motor area.
				Behavioral placebo analgesia correlates with reduced pain-related activity and increased frontoparietal activity, highlighting mechanisms of nociception, affect, and decision-making in pain.
				Significant between-study heterogeneity suggests variability in cerebral mechanisms across studies.
				Limitations:
				High between-study heterogeneity limits the ability to generalize findings across placebo analgesia contexts.
				Focuses on healthy participants; results may not directly translate to clinical populations with chronic pain.
				While robust at the neural level, behavioral and psychological interpretations of findings are limited.
				Excluded eight eligible studies due to lack of participant-level data, potentially introducing selection bias.
Bieniek, Bąbel (2023) [86]	Experimental study	Placebo hypoalgesia induced through operant conditioning using verbal, social, and token-based rewards and punishers	Healthy volunteers	Outcomes:
				Placebo hypoalgesia was successfully induced in groups with social and token-based reinforcement, but not with verbal reinforcement alone.
				Expectations of pain mediated the hypoalgesic effect, suggesting cognitive involvement.
				The number of reinforcers received predicted the magnitude of hypoalgesia, highlighting the role of conditioning intensity.
				Findings suggest token-based and social consequences may optimize pain management interventions.
				Limitations:
				Focused on healthy participants, limiting generalizability to clinical populations with chronic pain.
				Did not evaluate the long-term stability of placebo hypoalgesia effects.
				The study lacked diversity in participant demographics, potentially influencing the broader applicability of findings.
				While results highlight conditioning effects, their translation to clinical practice requires further investigation.
Testa et al. (2023) [84]	Book chapter/review	Management of cognitive, relational, and environmental contextual factors to optimize placebo effects and minimize nocebo effects in clinical practice	General population	Outcomes:
				Contextual factors, including beliefs, expectations, and therapeutic relationships, significantly enhance the outcomes of evidence-based treatments.
				Effective management of negative mindsets through empathic relationships can improve patient experience.
				Clinician’s attitude and skills in addressing contextual effects add measurable value to the therapeutic process.
				Limitations:
				The review provides theoretical guidance but lacks empirical validation of specific strategies for managing contextual factors.
				Generalized conclusions may not apply across all patient populations or clinical settings.
				Limited discussion of practical implementation challenges in clinical practice.
Colloca et al. (2023) [85]	Book chapter/review	Cultural influences on placebo and nocebo responses, including beliefs, rituals, and healthcare relationships	General population	Outcomes:
				Cultural beliefs, norms, and values shape treatment expectations and responses to placebo and nocebo effects.
				Physical and aesthetic preferences, influenced by culture, affect the perceived efficacy of treatments.
				Spiritual and religious beliefs impact coping strategies and treatment responses.
				Rituals and healthcare provider-patient dynamics (e.g., verbal and nonverbal cues) are critical in shaping placebo/nocebo responses.
				Limitations:
				The review is theoretical and lacks empirical data directly validating the role of cultural factors in placebo/nocebo responses.
				Generalizations are based on broad cultural concepts, which may not capture specific individual or subgroup variations.
				Limited exploration of how cultural factors interact with biological or psychological mechanisms.
Crawford et al. (2023) [144]	Experimental study	Brain mechanisms of placebo analgesia	Healthy volunteers	Outcomes:
				No significant differences in gamma-aminobutyric acid (GABA) or other metabolites between placebo responders and non-responders in the right DLPFC.
				Identified an inverse relationship between glutamate levels and pain rating variability during conditioning.
				Demonstrated altered functional connectivity between the DLPFC and midbrain periaqueductal gray (PAG) during placebo analgesia.
				Highlighted the role of the DLPFC in shaping stimulus-response relationships during conditioning.
				Limitations:
				The study was conducted on healthy individuals, limiting its applicability to clinical populations.
				The small sample size (38 participants) reduces the generalizability of findings.
				The study focuses only on acute pain scenarios, limiting its relevance to chronic pain contexts.
				Correlational nature of findings does not establish causation between DLPFC activity and placebo response.
Hartmann et al. (2023) [81]	Experimental study	Empathy-related psychological and structural brain differences between placebo analgesia responders and non-responders	Healthy volunteers	Outcomes:
				Placebo analgesia responders exhibited higher helping behavior and lower psychopathic traits compared to non-responders.
				Responders showed greater pain-related empathic concern.
				Structural brain differences: non-responders had increased gray matter volume in areas like the left inferior temporal and parietal supramarginal cortical regions and increased cortical surface area in the bilateral middle temporal cortex.
				Limitations:
				Uncorrected results in some analyses may lead to overestimated conclusions.
				Focus on a relatively narrow trait-based classification (e.g., empathy, psychopathy) without comprehensive exploration of other individual differences.
				Study paradigm and setting could influence outcomes, suggesting that contextual factors were not fully controlled for.
Meeuwis et al. (2023) [87]	Systematic Review and Meta-analysis	The effect of observational learning on placebo hypoalgesia and nocebo hyperalgesia	Healthy volunteers	Outcomes:
				Invest Observational learning (OL) had a small-to-medium effect on pain ratings (SMD = 0.44).
				- OL had a large effect on pain expectancy (SMD = 1.11).
				Empathic concern of the observer was positively correlated with the magnitude of placebo/nocebo effects (r = 0.14).
				Type of observation (in-person vs. videotaped) influenced the effect size (*p* < 0.01).
				Limitations:
				Moderate heterogeneity across studies.
				No clear clinical application of findings in chronic pain populations.
				Lack of placebo type modulation in the results (*p* = 0.23), suggesting further research is needed to clarify its role.
				Limited exploration of other empathy-related factors beyond empathic concern.
Rossettini et al. (2023) [138]	State of the art review	Overview of placebo and nocebo effects in experimental and chronic pain	Healthy volunteers and chronic pain patients	Outcomes:
				Strong evidence that placebo and nocebo effects are influenced by the psychosocial context.
				Psychological mechanisms and neurobiological/genetic determinants of placebo and nocebo effects are detailed.
				Differences in the occurrence of these effects between experimental settings (healthy participants) and clinical settings (chronic pain patients).
				Emphasizes the heterogeneity of pain in chronic patients affecting the magnitude of these effects.
				Limitations:
				Heterogeneity of pain in chronic patients makes results difficult to generalize.
				No unified results on the magnitude and occurrence of placebo/nocebo effects in chronic pain patients.
				Lacks specific experimental data to validate the proposed mechanisms in clinical settings.
				Calls for future research to address these gaps and improve the understanding of contextual factors.
Caliskan et al. (2024) [97]	Clinical update Review	Focus on treatment expectations, placebo/nocebo effects, and contextual factors	Patients in clinical settings, with an emphasis on pain management	Outcomes:
				Treatment expectations significantly influence treatment outcomes, acting as powerful modulators of health outcomes.
				Contextual factors that modify expectations can improve therapy success.
				Placebo analgesia and nocebo hyperalgesia are key mechanisms in the management of pain, with the expectations contributing to the overall treatment success.
				Further research is needed to personalize treatment strategies based on individual patient expectations.
				Limitations:
				The article is a clinical update and relies on existing evidence, with limited experimental data.
				It discusses variability in placebo/nocebo responses but does not identify clear predictors for individual responses.
				Calls for future research to explore personalized approaches to modulating treatment expectations.
				Does not address all clinical conditions in depth beyond pain.
Pedersen et al. (2024) [96]	Systematic review and meta-analysis	Focus on placebo effects, specific treatment effects, and changes observed without treatment in interventions for chronic nonspecific low back pain (NSLBP)	Adults with chronic nonspecific low back pain (NSLBP)	Outcomes:
				Approximately half of the overall treatment effect in conservative interventions for chronic NSLBP is attributed to changes observed without treatment, with smaller contributions from specific treatment and placebo effects.
				For pain intensity, 33% is attributed to specific treatment effects, 18% to placebo effects, and 49% to no-treatment changes.
				For physical function and HRQoL, 53% and 48% of the effect, respectively, is due to no treatment changes.
				Limitations:
				Low certainty of evidence, suggesting that the true effects might differ significantly from the reported estimates.
				The study is focused on conservative and passive interventions, which limits the applicability to other treatment types.
				The findings are based on short-term treatment effects and may not reflect long-term outcomes.
Saueressig et al. (2024) [46]	Review and methodological analysis	Focus on the methods used to quantify contextual effects in clinical care, particularly in placebo-controlled studies	N/A	Outcomes:
				The study critiques existing methods for quantifying contextual effects and proposes that the most effective method is comparing a placebo group with a non-treated control group.
				Other methods (such as the placebo control arm alone or proportional contextual effect calculation) are deemed inappropriate.
				This paper aims to provide guidance on best practices for estimating contextual effects in clinical research.
				Limitations:
				The review lacks empirical data as it is a methodological analysis, meaning it does not directly address clinical outcomes or interventions.
				It focuses only on theoretical frameworks and does not provide practical examples or real-world clinical applications.
				The effectiveness of the proposed method has not been fully tested or validated in diverse clinical settings.

## Appendix D. Effects and Potential Mechanisms of Osteopathy and Chiropractic in Musculoskeletal Pain: Cognitive-Mediated Effects and Bias in Osteopathic and Chiropractic Practices for Musculoskeletal Care

**Author (Year)**	**Study Type**	**Psychological Elements of the CAMs**	**Population**	**Outcomes and Limitations**
Forer (1949) [115]	Experimental	Personal validation fallacy	College students	Outcomes:
				Demonstrated how people tend to accept vague, general personality descriptions as accurate.
				Limitations:
				Limited sample, potential experimenter bias.
Beyerstein (2001) [124]	Review	Reasoning errors in alternative medicine	General population	Outcomes:
				Identified common logical fallacies in CAM beliefs.
				Limitations:
				Lack of empirical data.
Kaptchuk (2002) [116]	Review	Placebo effect in CAM	General population	Outcomes:
				Discussed potential clinical significance of healing rituals.
				Limitations:
				Lack of original data.
Winslow, Shapiro (2002) [108]	Cross-sectional survey	Physicians’ attitudes towards CAM education	American physicians	Outcomes:
				Physicians want more CAM education to better communicate with patients.
				Limitations:
				Potential response bias.
Klein, Helweg-Larsen (2002) [111]	Meta-analysis	Perceived control and optimistic bias	General population	Outcomes:
				Positive correlation between perceived control and optimistic bias.
				Limitations:
				Heterogeneity in included studies.
				The findings may not be generalizable to the use of CAM.
Honda et al. (2005) [99]	Cross-sectional survey	Personality, coping strategies, and social support in CAM use	American adults	Outcomes:
				Personality traits, coping strategies and social support influence CAM use.
				Limitations:
				Self-reported data, potential recall bias.
Singh et al. (2005) [113]	Qualitative study is based on in-person interviews	Motivation for CAM use	Men with prostate cancer	Outcomes:
				Identified various motivations for CAM use, including hope and empowerment.
				Limitations:
				Small sample size.
				Limited generalizability to musculoskeletal care.
Shih et al. (2009) [114]	Cross-sectional survey	CAM usage patterns	Singaporean adult cancer patients	Outcomes:
				High prevalence of CAM use, influenced by cultural factors.
				Limitations:
				Single-center study.
				Potential selection bias.
				Limited generalizability to musculoskeletal care.
Sperber (2010) [120]	Theoretical review	The “Guru Effect” in alternative beliefs	N/A	Outcomes:
				Proposed mechanism for why people trust incomprehensible ideas from perceived authorities.
				Limitations:
				Lack of empirical testing.
Wolfe, Michaud (2010) [122]	Observational study	Hawthorne effect in clinical trials	Patients with rheumatoid arthritis (RA)	Outcomes:
				Patients showed improved outcomes during the screening process before receiving any treatment.
				This effect led to an overestimation of treatment efficacy in clinical trials.
				Limitations:
				Study based on observational data, which may limit causal inferences.
				Potential confounders are not fully controlled.
				Generalizability to other conditions or trial designs may be limited.
Berthelot et al. (2011) [121]	Commentary	Hawthorne effect vs placebo effect	N/A	Outcomes:
				Argued Hawthorne effect may be stronger than placebo in some cases.
				Limitations:
				Limited empirical evidence presented.
Walach (2013) [90]	Book chapter/review	Placebo effects in CAM	General population	Outcomes:
				Discusses the role of placebo effects in CAM, suggesting that these effects may be particularly strong in CAM due to the holistic approach and strong therapeutic relationships.
				Proposes that CAM might trigger self-healing responses through various contextual and psychological factors.
				Limitations:
				Not peer-reviewed research.
				May lack the rigorous methodology of a systematic review or meta-analysis.
				The generalizability of the conclusions may be limited due to the diverse nature of CAM practices.
Benedetti et al. (2013) [98]	Experimental	Pain perception and opioid/cannabinoid systems	Healthy volunteers	Outcomes:
				Changing pain meaning from negative to positive activates opioid and cannabinoid systems.
				Limitations:
				Small sample size.
				laboratory setting.
Yarritu, Matute (2015) [104]	Experimental	Causal illusion in health beliefs	University students	Outcomes:
				Prior knowledge can induce an illusion of causality through biased behavior.
				Limitations:
				Artificial laboratory task.
Blanco (2017) [102]	Book chapter/Review	Cognitive bias	General population	Outcomes:
				Defined and described various cognitive biases.
				Limitations:
				Not original research.
				Not peer-reviewed research.
Stub et al. (2017) [118]	Qualitative interviews	Complementary therapists’ reflections on practice	Norwegian CAM practitioners	Outcomes:
				Therapists often refer to “patient healing power” as placebo effect.
				Limitations:
				Small sample.
				Potential social desirability bias.
Galbraith et al. (2018) [112]	Systematic review	Traits and cognitions associated with CAM use/belief	CAMs user	Outcomes:
				Identified personality traits and cognitive styles linked to CAM use.
				Limitations:
				Heterogeneity in included studies.
Garrett et al. (2019) [119]	Mixed methods	Perceptions of internet-based health scams	UK adults	Outcomes:
				Identified factors promoting engagement with online health scams.
				Limitations:
				Potential selection bias in online sample.
Moreno Castro et al. (2019) [101]	Qualitative research methods	Influences on perception of pseudo-therapies	Spanish population	Outcomes:
				Media, social circles, and education influence pseudo-therapy beliefs.
				Limitations:
				Self-reported data, potential social desirability bias.
Chow et al. (2021) [105]	Experimental	Causal relationships in pseudoscientific health beliefs	University students	Outcomes:
				Perceived frequency of causal relationships influences pseudoscientific beliefs.
				Limitations:
				Artificial laboratory task.
Rodríguez-Ferreiro et al. (2021) [106]	Experimental	Evidential criteria in pseudoscience believers	Spanish adults	Outcomes:
				Pseudoscience believers have lower evidential criteria.
				Limitations:
				The online sample may not be representative of the general population.
				Self-reported measurements may be subject to bias.
				The study’s correlational nature limits causal inferences about the relationship between evidential criteria and pseudoscientific beliefs.
Davies et al. (2022) [117]	Systematic review	Knowledge used in CAM consultations	Physicians and patients	Outcomes:
				Classified types of knowledge used in CAM practice.
				Limitations:
				Heterogeneity in included studies.
Esteves et al. (2022) [100]	Theoretical paper	Osteopathic care as enactive inference	General population	Outcomes:
				Proposed theoretical framework for osteopathic practice.
				Limitations:
				Lack of empirical testing.
Garcia-Arch et al. (2022) [107]	Experimental	Expert feedback on pseudoscientific beliefs	Spanish adults	Outcomes:
				Expert feedback can increase acceptance of health-related pseudoscientific beliefs.
				Limitations:
				Online sample.
				Artificial task.
García-Arch et al. (2022) [109]	Correlational	Prediction of pseudoscience acceptance	Spanish adults	Outcomes:
				Information interpretation and individual differences predict pseudoscience acceptance.
				Limitations:
				Cross-sectional design, self-reported data.
Piñeiro Pérez et al. (2022) [110]	Cross-sectional survey	Pediatricians’ knowledge and use of CAM	Spanish pediatricians	Outcomes:
				Identified gaps in CAM knowledge among pediatricians.
				Limitations:
				Potential response bias.
Segovia et al. (2022) [9]	Cross-sectional survey	Trust and belief in pseudotherapies	Spanish adults	Outcomes:
				Pseudotherapy use is associated with trust in efficacy rather than belief in scientific validity.
				Limitations:
				Self-reported data.
				Potential social desirability bias.
Torres et al. (2022) [103]	Experimental	Causal illusion in pseudoscientific beliefs	Spanish university students	Outcomes:
				Information interpretation and search strategies influence causal illusions.
				Limitations:
				It does not allow us to know the causality between the illusions of causality and the tendency to maintain unjustified beliefs.
				There may be variables that are not controlled.
Vicente et al. (2023) [125]	Experimental	Prior beliefs’ influence on judgments of medicine effectiveness	University students	Outcomes:
				Prior beliefs influence judgments about both alternative and scientific medicine.
				Limitations:
				The online sample may not be representative, which prevents generalization of the results.
				Potential social desirability bias.
				The correlational nature of the study limits causal inferences.
				The study is based on hypothetical scenarios, which may not fully reflect how people would make decisions in real health situations.
				The study cannot fully control for other factors that might influence judgments about the effectiveness of treatments.
Neogi, Colloca (2023) [123]	Narrative review	Placebo effects in osteoarthritis	Patients with osteoarthritis	Outcomes:
				Placebo effects contribute significantly to pain relief in osteoarthritis.
				These effects are mediated by psychological factors and neurobiological mechanisms.
				Placebo responses may be enhanced by several factors, including the therapeutic encounter, treatment characteristics, and individual patient factors.
				It is suggested that understanding and harnessing placebo effects could improve clinical outcomes and drug development in osteoarthritis.
				Limitations:
				The review is based on existing literature, which may have variable quality and methodologies.
				Generalizability of the findings to all patients with osteoarthritis may be limited.
				The review does not provide new empirical data.
				The long-term effects of placebo responses in osteoarthritis are not well established.

## Appendix E. Effects and Potential Mechanisms of Osteopathy and Chiropractic in Musculoskeletal Pain: The Effects Mediated by Context in Osteopathic and Chiropractic Practices for Musculoskeletal Care

**Author (Year)**	**Study Type**	**Intervention/Contextual Focus**	**Population**	**Outcomes and Limitations**
Kaptchuk (2002) [116]	Narrative review	Placebo Effect in CAM	General population	Outcomes:
				Discusses how ritualistic and symbolic aspects of alternative medicine can evoke clinically significant placebo responses.
				Limitations:
				Primarily theoretical; lacks empirical data to substantiate claims.
Paterson (2005) [133]	Narrative review	Placebo effect in acupuncture	Acupuncture patients	Outcomes:
				Distinguishes between characteristic and incidental (placebo) effects in acupuncture efficacy.
				Limitations:
				Lacks experimental data, limited generalizability beyond acupuncture.
Linde et al. (2005) [134]	Randomized Controlled Trial	Acupuncture	Migraine patients	Outcomes:
				The possible benefits of acupuncture may be due to factors other than those derived from the needling.
				Limitations:
				Lack of significant difference with control group suggests influence of non-specific factors.
Diener et al. (2006) [135]	RCT	Acupuncture	Migraine patients	Outcomes:
				Treatment outcomes for migraine did not differ significantly between verum acupuncture, sham acupuncture, and standard therapy groups, suggesting a strong influence of contextual factors
				Limitations:
				High dropout rate in the standard therapy group (106 patients) may have affected group comparability.
				Inability to blind participants to standard drug therapy could have influenced patient-reported outcomes.
				The study design did not allow for isolation of specific contextual factors from overall treatment effects.
Fulda et al. (2007) [128]	Pilot study	Osteopathic Manipulative Treatment (OMT)	Low back pain patients	Outcomes:
				Positive expectations can influence perceived efficacy, even in placebo treatments.
				Limitations:
				Small sample size limits generalizability; preliminary findings lack statistical power.
				Lack of control groups reduces the ability to isolate the impact of expectations.
Meissner et al. (2013) [130]	Systematic review	Placebo in migraine prophylaxis	Migraine patients	Outcomes:
				Efficacy among placebo treatments in preventing migraine.
				Limitations:
				Heterogeneity of included studies may affect consistency of conclusions.
Calpin et al. (2017) [127]	Comparative retrospective study.	Chronic pain management	Patients with chronic pain	Outcomes:
				Discrepancies in expectations were noted, with significant effects from patient characteristics like age, gender, and sleep quality on expectations. The study highlights the need to align expectations for better outcomes.
				Limitations:
				Small sample of physicians limits generalization.
				Based on descriptive comparisons only.
				Lack of follow-up after consultation.
				Possible misinterpretation of free responses.
Rossettini et al. (2018) [126]	Narrative review.	Placebo/nocebo in MSK care	Patients with musculoskeletal pain	Outcomes:
				Highlights influence of contextual factors on placebo and nocebo effects.
				Limitations:
				Lacks comprehensive analysis of primary data; broad generalizations may limit applicability.
Thomson et al. (2021) [95]	Clinical Commentary	Placebo/Contextual factors	General MSK care	Outcomes:
				Emphasizes the importance of contextual factors in enhancing treatment effects.
				Limitations:
				Lacks original data; primarily theoretical commentary.
Tsutsumi et al. (2023) [136]	Meta-epidemiological study	Contextual effects in general medicine	Data from Cochrane reviews	Outcomes:
				Estimates significant portion of medical treatment results attributable to contextual and placebo effects.
				Limitations:
				Focus on general medicine may limit direct applicability to musculoskeletal care.
Nim et al. (2025) [131]	Systematic review with network meta-analysis	Spinal manipulative therapy (SMT) application procedures	Adults with spine pain	Outcomes:
				Most SMT procedures were slightly more effective than other treatments; a general and non-specific SMT approach had the highest probability of achieving the largest effects.
				Limitations:
				Differences between SMT approaches were small and not clinically relevant; evidence was of low to very low certainty due to heterogeneity, bias, and lack of direct comparisons

## References

[1] Kemppainen L.M., Kemppainen T.T., Reippainen J.A., Salmenniemi S.T., Vuolanto P.H. (2018). Use of Complementary and Alternative Medicine in Europe: Health-Related and Sociodemographic Determinants. Scand. J. Public Health.

[2] Møller S.R., Ekholm O., Christensen A.I. (2024). Trends in the Use of Complementary and Alternative Medicine between 1987 and 2021 in Denmark. BMC Complement. Med. Ther..

[3] Bishop F.L., Yardley L., Lewith G.T. (2007). A Systematic Review of Beliefs Involved in the Use of Complementary and Alternative Medicine. J. Health Psychol..

[4] Johnson P.J., Jou J., Rockwood T.H., Upchurch D.M. (2019). Perceived Benefits of Using Complementary and Alternative Medicine by Race/Ethnicity Among Midlife and Older Adults in the United States. J. Aging Health.

[5] Peltzer K., Pengpid S. (2018). Prevalence and Determinants of Traditional, Complementary and Alternative Medicine Provider Use among Adults from 32 Countries. Chin. J. Integr. Med..

[6] MacArtney J.I., Wahlberg A. (2014). The Problem of Complementary and Alternative Medicine Use Today: Eyes Half Closed?. Qual. Health Res..

[7] Hestbaeck L., Hartvigsen J., Christensen H., Werner V. (2023). Osteopathy and Chiropractic Treatment in Babies with Infantile Colic. Acta Paediatr..

[8] Lobera J., Rogero-García J.S.A. (2020). Homeopathy Determinants of Trust in Complementary and Alternative Medicine. Health Commun..

[9] Segovia G., Sanz-Barbero B. (2022). It Works for Me”: Pseudotherapy Use Is Associated With Trust in Their Efficacy Rather Than Belief in Their Scientific Validity. Int. J. Public Health.

[10] Sinclair A.J., Sturrock A., Davies B., Matharu M. (2015). Headache Management: Pharmacological Approaches. Pract. Neurol..

[11] Weatherall M. (2015). Drug Therapy in Headache. Clin. Med..

[12] Lipton R.B., Serrano D., Holland S., Fanning K.M., Reed M.L., Buse D.C. (2013). Barriers to the Diagnosis and Treatment of Migraine: Effects of Sex, Income, and Headache Features. Headache.

[13] Rossi P., Lorenzo G., Malpezzi M., Faroni J., Cesarino F., Lorenzo C., Nappi G. (2005). Prevalence, Pattern and Predictors of Use of Complementary and Alternative Medicine (CAM) in Migraine Patients Attending a Headache Clinic in Italy. Cephalalgia.

[14] Moore C.S., Sibbritt D.W., Adams J. (2017). A Critical Review of Manual Therapy Use for Headache Disorders: Prevalence, Profiles, Motivations, Communication and Self-Reported Effectiveness. BMC Neurol..

[15] Bustins G.A., Plaza P.-V.L., Carvajal S.R. (2018). Profile of Osteopathic Practice in Spain: Results from a Standardized Data Collection Study. BMC Complement. Altern. Med..

[16] WMA Declaration of Cordoba on Patient-Physician Relationship. https://www.wma.net/policies-post/wma-declaration-of-cordoba-on-patient-physician-relationship/.

[17] Danell J.A.B., Danell R., Vuolanto P. (2020). Fifty Years of Complementary and Alternative Medicine (CAM)—A Bibliometric Analysis of Publication Activity and General Content of the Publications. J. Sci. Res..

[18] N.I.H. National Center for Complementary and Integrative Health (NCCIH) (2021). Obtenido de Complementary, Alternative, or Integrative Health: What’s In a Name?.

[19] Pettman E. (2007). A History of Manipulative Therapy. J. Man. Manip. Ther..

[20] Federspil G., Vettor R. (2000). Can scientific medicine incorporate alternative medicine?. J. Altern. Complement. Med..

[21] Rajtmajer S.M., Errington T.M., Hillary F.G. (2022). How Failure to Falsify in High-Volume Science Contributes to the Replication Crisis. eLife.

[22] Palsson T.S., Gibson W., Darlow B., Bunzli S., Lehman G., Rabey M., Moloney N., Vaegter H.B., Bagg M.K., Travers M. (2019). Changing the Narrative in Diagnosis and Management of Pain in the Sacroiliac Joint Area. Phys. Ther..

[23] Tascilar M., Jong F.A., Verweij J., Mathijssen R.H.J. (2006). Complementary and Alternative Medicine during Cancer Treatment: Beyond Innocence. Oncologist.

[24] Gasparyan A.Y., Ayvazyan L., Blackmore H., Kitas G.D. (2011). Writing a Narrative Biomedical Review: Considerations for Authors, Peer Reviewers, and Editors. Rheumatol. Int..

[25] Koterov A.U. (2021). Hill’s “Biological Plausibility” Criterion: Integration of Data from Various Disciplines for Epidemiology and Radiation Epidemiology. Biol. Bull. Russ. Acad. Sci..

[26] Roberts A., Harris K., Outen B., Bukvic A., Smith B., Schultz A., Bergman S., Mondal D. (2022). Osteopathic Manipulative Medicine: A Brief Review of the Hands-On Treatment Approaches and Their Therapeutic Uses. Medicines.

[27] Thomson O., MacMillan A. (2023). What’s Wrong with Osteopathy?. Int. J. Osteopath. Med..

[28] Bonanno M., Papa G.A., Ruffoni P., Catalioto E., Luca R., Maggio M.G., Calabrò R.S. (2024). The Effects of Osteopathic Manipulative Treatment on Brain Activity: A Scoping Review of MRI and EEG Studies. Healthcare.

[29] Ceballos-Laita L., Jiménez-Del-Barrio S., Carrasco-Uribarren A., Medrano-De-La-Fuente R., Robles-Pérez R., Ernst E. (2024). Is Osteopathic Manipulative Treatment Clinically Superior to Sham or Placebo for Patients with Neck or Low-Back Pain? A Systematic Review with Meta-Analysis. Diseases.

[30] Bordoni B., Escher A.R. (2023). The Osteopath’s Imprint: Osteopathic Medicine Under the Nanoscopic Lens. Cureus.

[31] Crow W.T., King H.H., Patterson R.M., Giuliano V. (2009). Assessment of Calvarial Structure Motion by MRI. Osteopath. Med. Prim. Care.

[32] Requena-García J., García-Nieto E., Varillas-Delgado D. (2021). Objectivation of an Educational Model in Cranial Osteopathy Based on Experience. Medicina.

[33] Horton R. (2015). The Anatomy, Biological Plausibility and Efficacy of Visceral Mobilization in the Treatment of Pelvic Floor Dysfunction. J. Pelvic Obstet. Gynaecol. Physiother..

[34] Cazzola D., Alberti G., Ongaro L., Minetti A.E. (2014). The Vertical Excursion of the Body Visceral Mass during Vertical Jumps Is Affected by Specific Respiratory Maneuver. Hum. Mov. Sci..

[35] Hidalgo D.F., MacMillan A., Thomson O.P. (2024). It’s All Connected, so It All Matters’’—The Fallacy of Osteopathic Anatomical Possibilism. Int. J. Osteopath. Med..

[36] Guillaud A., Darbois N., Monvoisin R., Pinsault N. (2018). Reliability of Diagnosis and Clinical Efficacy of Visceral Osteopathy: A Systematic Review. BMC Complement. Altern. Med..

[37] Consorti G., Castagna C., Tramontano M., Longobardi M., Castagna P., Lernia D., Lunghi C. (2023). Reconceptualizing Somatic Dysfunction in the Light of a Neuroaesthetic Enactive Paradigm. Healthcare.

[38] Côté P., Bussieres A., Cassidy J.D., Hartvigsen J., Kawchuk G.N., Leboeuf-Yde C., Mior S., Schneider M., Aillet L., Ammendolia C. (2020). A United Statement of the Global Chiropractic Research Community against the Pseudoscientific Claim That Chiropractic Care Boosts Immunity. Chiropr. Man. Ther..

[39] Haas A., Chung J., Kent C., Mills B., McCoy M. (2024). Vertebral Subluxation and Systems Biology: An Integrative Review Exploring the Salutogenic Influence of Chiropractic Care on the Neuroendocrine-Immune System. Cureus.

[40] Homola S. (2013). Pseudoscience in the Use of Manipulation by Chiropractors. Focus Altern. Complement. Ther..

[41] Homola S. (2016). Pediatric Chiropractic Care: The Subluxation Question And Referral Risk. Bioethics.

[42] Mirtz T.A., Morgan L., Wyatt L.H., Greene L. (2009). An Epidemiological Examination of the Subluxation Construct Using Hill’s Criteria of Causation. Chiropr. Osteopat..

[43] Nim C.G., Downie A., O’neill S., Kawchuk G.N., Perle S.M., Leboeuf-Yde C. (2021). The Importance of Selecting the Correct Site to Apply Spinal Manipulation When Treating Spinal Pain: Myth or Reality? A Systematic Review. Sci. Rep..

[44] Johansen M.K., Osman M. (2020). Coincidence Judgment in Causal Reasoning: How Coincidental Is This?. Cogn. Psychol..

[45] Ezzatvar Y., Dueñas L., Balasch-Bernat M., Lluch-Girbés E., Rossettini G. (2024). Which Portion of Physiotherapy Treatments’ Effect Is Not Attributable to the Specific Effects in People with Musculoskeletal Pain? A Meta-Analysis of Randomized Placebo-Controlled Trials. J. Orthop. Sports Phys. Ther..

[46] Saueressig T., Pedder H., Owen P.J., Belavy D.L. (2024). Contextual Effects: How to, and How Not to, Quantify Them. BMC Med. Res. Methodol..

[47] Franke H., Franke J.-D., Fryer G. (2014). Osteopathic Manipulative Treatment for Nonspecific Low Back Pain: A Systematic Review and Meta-Analysis. BMC Musculoskelet. Disord..

[48] Rehman Y., Ferguson H., Bozek A., Blair J., Allison A., Johnston R. (2020). Osteopathic Manual Treatment for Pain Severity, Functional Improvement, and Return to Work in Patients With Chronic Pain. J. Am. Osteopath. Assoc..

[49] Farra F.D., Risio R.G., Vismara L., Bergna A. (2021). Effectiveness of Osteopathic Interventions in Chronic Non-Specific Low Back Pain: A Systematic Review and Meta-Analysis. Complement. Ther. Med..

[50] Farra F.D., Buffone F., Risio R.G., Tarantino A.G., Vismara L., Bergna A. (2022). Effectiveness of Osteopathic Interventions in Patients with Non-Specific Neck Pain: A Systematic Review and Meta-Analysis. Complement. Ther. Clin. Pract..

[51] Lotfi C., Blair J., Jumrukovska A., Grubb M., Glidden E., Toldi J. (2023). Effectiveness of Osteopathic Manipulative Treatment in Treating Symptoms of Irritable Bowel Syndrome: A Literature Review. Cureus.

[52] Ernst E. (2012). Craniosacral Therapy: A Systematic Review of the Clinical Evidence. Focus. Altern. Complement. Ther..

[53] Guillaud A., Darbois N., Monvoisin R., Pinsault N. (2016). Reliability of Diagnosis and Clinical Efficacy of Cranial Osteopathy: A Systematic Review. PLoS ONE.

[54] Silva F.C., Vieira L.S., Santos L.V., Gaudreault N., Cruvinel-Júnior R.H., Santos G.M. (2023). Effectiveness of Visceral Fascial Therapy Targeting Visceral Dysfunctions Outcome: Systematic Review of Randomized Controlled Trials. BMC Complement. Med. Ther..

[55] Buffone F., Tarantino A.G., Belloni F., Spadafora A., Bolzoni G., Bruini I., Bergna A., Vismara L. (2023). Effectiveness of Osteopathic Manipulative Treatment in Adults with Irritable Bowel Syndrome: A Systematic Review and Meta-Analysis. Healthcare.

[56] Ceballos-Laita L., Ernst E., Carrasco-Uribarren A., Cabanillas-Barea S., Esteban-Pérez J., Jiménez-Del-Barrio S. (2024). Is Craniosacral Therapy Effective? A Systematic Review and Meta-Analysis. Healthcare.

[57] Ceballos-Laita L., Ernst E., Carrasco-Uribarren A., Esteban-Tarcaya G., Mamud-Meroni L., Jiménez-del-Barrio S. (2024). Is Visceral Osteopathy Therapy Effective? A Systematic Review and Meta-Analysis. Int. J. Osteopath. Med..

[58] Ceballos-Laita L., Mingo-Gomez M.T., Medrano-de-la-Fuente R., Hernando-Garijo I., Jimenez-del-Barrio S. (2023). The Effectiveness of Visceral Osteopathy in Pain, Disability, and Physical Function in Patients with Low-Back Pain. A Systematic Review and Meta-Analysis. Explore.

[59] Ajimsha M., Daniel B., Chithra S. (2014). Effectiveness of Myofascial Release in the Management of Chronic Low Back Pain in Nursing Professionals. J. Bodyw. Mov. Ther..

[60] Arguisuelas M.D., Lisón J.F., Sánchez-Zuriaga D., Martínez-Hurtado I., Doménech-Fernández J. (2017). Effects of Myofascial Release in Nonspecific Chronic Low Back Pain. Spine.

[61] Nguyen C., Boutron I., Zegarra-Parodi R., Baron G., Alami S., Sanchez K., Daste C., Boisson M., Fabre L., Krief P. (2021). Effect of Osteopathic Manipulative Treatment vs. Sham Treatment on Activity Limitations in Patients With Nonspecific Subacute and Chronic Low Back Pain: A Randomized Clinical Trial. JAMA Intern. Med..

[62] Rotter G., Fernholz I., Binting S., Keller T., Roll S., Kass B., Reinhold T., Willich S.N., Schmidt A., Brinkhaus B. (2020). The Effect of Osteopathic Medicine on Pain in Musicians with Nonspecific Chronic Neck Pain: A Randomized Controlled Trial. Ther. Adv. Musculoskelet. Dis..

[63] Gross A., Miller J., D’sylva J., Burnie S.J., Goldsmith C.H., Graham N., Haines T., Brønfort G., Hoving J.L. (2010). Manipulation or Mobilisation for Neck Pain: A Cochrane Review. Man. Ther..

[64] Walker B.F., French S.D., Grant W., Green S. (2010). Combined Chiropractic Interventions for Low-Back Pain. Cochrane Database Syst. Rev..

[65] Rubinstein S.M., Zoete A., Middelkoop M., Assendelft W.J.J., Boer M.R., Tulder M.W. (2019). Benefits and Harms of Spinal Manipulative Therapy for the Treatment of Chronic Low Back Pain: Systematic Review and Meta-Analysis of Randomised Controlled Trials. BMJ.

[66] Gevers-Montoro C., Provencher B., Descarreaux M., Mues A.O., Piché M. (2021). Clinical Effectiveness and Efficacy of Chiropractic Spinal Manipulation for Spine Pain. Front. Pain Res..

[67] Ernst E. (2012). Chiropractic Spinal Manipulation: What Does the “Best” Evidence Show? Focus Altern. Complement. Ther..

[68] Ernst E. (2008). Chiropractic: A Critical Evaluation. J. Pain Symptom Manag..

[69] Kranenburg H., Schmitt M., Puentedura E., Luijckx G., Schans C. (2017). Adverse Events Associated with the Use of Cervical Spine Manipulation or Mobilization and Patient Characteristics: A Systematic Review. Musculoskelet. Sci. Pract..

[70] Hohenschurz-Schmidt D., Thomson O.P., Rossettini G., Miciak M., Newell D., Roberts L., Vase L., Draper-Rodi J. (2022). Avoiding Nocebo and Other Undesirable Effects in Chiropractic, Osteopathy and Physiotherapy: An Invitation to Reflect. Musculoskelet. Sci. Pract..

[71] Bishop M.D., Beneciuk J.M., George S.Z. (2011). Immediate Reduction in Temporal Sensory Summation after Thoracic Spinal Manipulation. Spine J..

[72] Aspinall S.L., Jacques A., Leboeuf-Yde C., Etherington S.J., Walker B.F. (2019). No Difference in Pressure Pain Threshold and Temporal Summation after Lumbar Spinal Manipulation Compared to Sham: A Randomised Controlled Trial in Adults with Low Back Pain. Musculoskelet. Sci. Pract..

[73] George S.Z., Bishop M.D., Bialosky J.E., Zeppieri G., Robinson M.E. (2006). Immediate Effects of Spinal Manipulation on Thermal Pain Sensitivity: An Experimental Study. BMC Musculoskelet. Disord..

[74] Nim C., Kawchuk G., Schiøttz-Christensen B., O’Neill S. (2020). The Effect on Clinical Outcomes When Targeting Spinal Manipulation at Stiffness or Pain Sensitivity: A Randomized Trial. Sci. Rep..

[75] Bialosky J.E., Beneciuk J.M., Bishop M.D., Coronado R.A., Penza C.W., Simon C.B., George S.Z. (2018). Unraveling the Mechanisms of Manual Therapy: Modeling an Approach. J. Orthop. Sports Phys. Ther..

[76] Farra F.D., Bergna A., Lunghi C., Bruini I., Galli M., Vismara L., Tramontano M. (2024). Reported Biological Effects Following Osteopathic Manipulative Treatment: A Comprehensive Mapping Review. Complement. Ther. Med..

[77] Colloca L., Klinger R., Flor H., Bingel U. (2013). Placebo Analgesia: Psychological and Neurobiological Mechanisms. Pain.

[78] Colloca L., Grillon C. (2014). Understanding Placebo and Nocebo Responses for Pain Management. Curr. Pain Headache.

[79] Shi Y., Cui S., Zeng Y., Huang S., Cai G., Yang J., Wu W. (2021). Brain Network to Placebo and Nocebo Responses in Acute Experimental Lower Back Pain: A Multivariate Granger Causality Analysis of FMRI Data. Front. Behav. Neurosci..

[80] Anderson S., Stebbins G.T. (2020). Determinants of Placebo Effects. Int. Rev. Neurobiol..

[81] Hartmann H., Banwinkler M., Riva F., Riva F., Lamm C. (2023). To Respond or Not to Respond: Exploring Empathy-Related Psychological and Structural Brain Differences between Placebo Analgesia Responders and Non-Responders. Front. Psychol..

[82] Blasini M., Peiris N., Wright T., Colloca L. (2018). The Role of Patient-Practitioner Relationships in Placebo and Nocebo Phenomena. Int. Rev. Neurobiol..

[83] Testa M., Rossettini G. (2016). Enhance Placebo, Avoid Nocebo: How Contextual Factors Affect Physiotherapy Outcomes. Man. Ther..

[84] Testa M., Rossettini G., Barbiani D., Miciak M., Colloca L. (2023). Management of Contextual Factors to Enhance Placebo and Minimize Nocebo Effects in Clinical Practice. Placebo Effects Through the Lens of Translational Research.

[85] Cundiff-O’Sullivan R., Oula D., Shafir R., Colloca L., Colloca L., Noel J., Franklin P.D., Seneviratne C. (2023). Cultural Influences on Placebo and Nocebo Effects. Placebo Effects Through the Lens of Translational Research.

[86] Bieniek H., Bąbel P. (2023). Placebo Hypoalgesia Induced by Operant Conditioning: A Comparative Study on the Effects of Verbal, Token-Based, and Social Rewards and Punishers. Sci. Rep..

[87] Meeuwis S.H., Wasylewski M.T., Bajcar E.A., Bieniek H., Adamczyk W.M., Honcharova S., Nardo M., Mazzoni G., Bąbel P. (2023). Learning Pain from Others: A Systematic Review and Meta-Analysis of Studies on Placebo Hypoalgesia and Nocebo Hyperalgesia Induced by Observational Learning. Pain.

[88] Eippert F., Bingel U., Schoell E.D., Yacubian J., Klinger R., Lorenz J., Büchel C. (2009). Activation of the Opioidergic Descending Pain Control System Underlies Placebo Analgesia. Neuron.

[89] Schweinhardt P., Seminowicz D.A., Jaeger E., Duncan G.H., Bushnell M.C. (2009). The Anatomy of the Mesolimbic Reward System: A Link between Personality and the Placebo Analgesic Response. J. Neurosci..

[90] Walach H., Colloca L., Flaten M.A., Meissner K. (2013). Placebo Effects in Complementary and Alternative Medicine: The Self-Healing Response. Placebo and Pain: From Bench to Bedside.

[91] Hróbjartsson A., Gøtzsche P.C. (2010). Placebo Interventions for All Clinical Conditions. Cochrane Database Syst. Rev..

[92] Ashar Y.K., Chang L.J., Wager T.D. (2017). Brain Mechanisms of the Placebo Effect: An Affective Appraisal Account. Annu. Rev. Clin. Psychol..

[93] Beedie C., Whyte G., Lane A.M., Cohen E., Raglin J., Hurst P., Coleman D., Foad A. (2018). Caution, This Treatment Is a Placebo. It Might Work, but It Might Not’: Why Emerging Mechanistic Evidence for Placebo Effects Does Not Legitimise Complementary and Alternative Medicines in Sport. Br. J. Sports Med..

[94] Wager T.D., Atlas L.Y. (2015). The Neuroscience of Placebo Effects: Connecting Context, Learning and Health. Nat. Rev. Neurosci..

[95] Thomson O., Rossettini G. (2021). Don’t Focus on the Finger, Look at the Moon’—The Importance of Contextual Factors for Clinical Practice and Research. Int. J. Osteopath. Med..

[96] Pedersen J.R., Strijkers R., Gerger H., Koes B., Chiarotto A. (2024). Clinical Improvements Due to Specific Effects and Placebo Effects in Conservative Interventions and Changes Observed with No Treatment in Randomized Controlled Trials of Patients with Chronic Nonspecific Low Back Pain: A Systematic Review and Meta-Analysi. Pain.

[97] Caliskan E.B., Bingel U., Kunkel A. (2024). Translating Knowledge on Placebo and Nocebo Effects into Clinical Practice. PAIN Rep..

[98] Benedetti F., Thoen W., Blanchard C., Vighetti S., Arduino C. (2013). Pain as a Reward: Changing the Meaning of Pain from Negative to Positive Co-Activates Opioid and Cannabinoid Systems. Pain.

[99] Honda K., Jacobson J.S. (2005). Use of Complementary and Alternative Medicine among United States Adults:The Influences of Personality, Coping Strategies, and Social Support. Prev. Med..

[100] Esteves J.E., Cerritelli F., Kim J., Friston K.J. (2022). Osteopathic Care as (En)Active Inference: A Theoretical Framework for Developing an Integrative Hypothesis in Osteopathy. Front. Psychol..

[101] Moreno-Castro C., Corell-Doménech M., Camano-Puig R. (2019). Which Has More Influence on Perception of Pseudo-Therapies: The Media’s Information, Friends or Acquaintances Opinion, or Educational Background?. Commun. Soc..

[102] Blanco F., Vonk J., Shackelford T. (2017). Cognitive Bias. Encyclopedia of Animal Cognition and Behavior.

[103] Torres M.N., Barberia I., Rodríguez-Ferreiro J. (2022). Causal Illusion in the Core of Pseudoscientific Beliefs: The Role of Information Interpretation and Search Strategies. PLoS ONE.

[104] Yarritu I., Matute H. (2015). Previous Knowledge Can Induce an Illusion of Causality through Actively Biasing Behavior. Front. Psychol..

[105] Chow J.Y.L., Colagiuri B., Rottman B.M., Goldwater M., Livesey E.J. (2021). Pseudoscientific Health Beliefs and the Perceived Frequency of Causal Relationships. Int. J. Environ. Res. Public Health.

[106] Rodríguez-Ferreiro J., Barberia I. (2021). Believers in Pseudoscience Present Lower Evidential Criteria. Sci. Rep..

[107] Garcia-Arch J., Barberia I., Rodríguez-Ferreiro J., Fuentemilla L. (2022). Authority Brings Responsibility: Feedback from Experts Promotes an Overweighting of Health-Related Pseudoscientific Beliefs. Int. J. Environ. Res. Public Health.

[108] Winslow L.C., Shapiro H. (2002). Physicians Want Education about Complementary and Alternative Medicine to Enhance Communication with Their Patients. Arch. Intern. Med..

[109] García-Arch J., Ballestero-Arnau M., Hoyas L.P., Giaiotti F. (2022). Disproven but Still Believed: The Role of Information and Individual Differences in the Prediction of Topic-Related Pseudoscience Acceptance. Appl. Cogn. Psychol..

[110] Pérez R.P., Cuadros E.N., García L.C., López I.D., Fernández R.E., Gil Lemus M.Á., Blanco S.M., Marrodán B.R., Calvo C. (2022). Results of a National Survey on Knowledge and Use of Complementary and Alternative Medicine by Paediatricians. An. Pediatr..

[111] Klein C.T., Helweg-Larsen M. (2002). Perceived Control and the Optimistic Bias: A Meta-Analytic Review. Psychol. Health.

[112] Galbraith N., Moss T., Galbraith V., Purewal S. (2018). A Systematic Review of the Traits and Cognitions Associated with Use of and Belief in Complementary and Alternative Medicine (CAM). Psychol. Health Med..

[113] Singh H., Maskarinec G., Shumay D.M. (2005). Understanding the Motivation for Conventional and Complementary/Alternative Medicine Use among Men with Prostate Cancer. Integr. Cancer Ther..

[114] Shih V., Chiang J.Y.L., Chan A. (2009). Complementary and Alternative Medicine (CAM) Usage in Singaporean Adult Cancer Patients. Ann. Oncol..

[115] Forer B.R. (1949). The Fallacy of Personal Validation: A Classroom Demonstration of Gullibility. J. Abnorm. Soc. Psychol..

[116] Kaptchuk T.J. (2002). The Placebo Effect in Alternative Medicine: Can the Performance of a Healing Ritual Have Clinical Significance? Ann. Intern. Med..

[117] Davies K., Heinsch M., Tickner C., Brosnan C., Steel A., Patel G., Marsh M. (2022). Classifying Knowledge Used in Complementary Medicine Consultations: A Qualitative Systematic Review. BMC Complement. Med. Ther..

[118] Stub T., Foss N., Liodden I. (2017). Placebo Effect Is Probably What We Refer to as Patient Healing Power”: A Qualitative Pilot Study Examining How Norwegian Complementary Therapists Reflect on Their Practice. BMC Complement. Altern. Med..

[119] Garrett B., Mallia E., Anthony J. (2019). Public Perceptions of Internet-Based Health Scams, and Factors That Promote Engagement with Them. Health Soc. Care Community.

[120] Sperber D. (2010). The Guru Effect. Rev. Philos. Psychol..

[121] Berthelot J.M., Le Goff B., Maugars Y. (2011). The Hawthorne Effect: Stronger than the Placebo Effect?. Jt. Bone Spine.

[122] Wolfe F., Michaud K. (2010). The Hawthorne Effect, Sponsored Trials, and the Overestimation of Treatment Effectiveness. J. Rheumatol..

[123] Neogi T., Colloca L. (2023). Placebo Effects in Osteoarthritis: Implications for Treatment and Drug Development. Nat. Rev. Rheumatol..

[124] Beyerstein B.L. (2001). Alternative Medicine and Common Errors of Reasoning. Acad. Med..

[125] Vicente L., Blanco F., Matute H. (2023). I Want to Believe: Prior Beliefs Influence Judgments about the Effectiveness of Both Alternative and Scientific Medicine. Judgm. Decis. Mak..

[126] Rossettini G., Carlino E., Testa M. (2018). Clinical Relevance of Contextual Factors as Triggers of Placebo and Nocebo Effects in Musculoskeletal Pain. BMC Musculoskelet. Disord..

[127] Calpin P., Imran A., Harmon D. (2017). A Comparison of Expectations of Physicians and Patients with Chronic Pain for Pain Clinic Visits. Pain Pract..

[128] Fulda K.G., Slicho T., Stoll S.T. (2007). Patient Expectations for Placebo Treatments Commonly Used in Osteopathic Manipulative Treatment (OMT) Clinical Trials: A Pilot Study. Osteopath. Med. Prim. Care.

[129] Bialosky J.E., Bishop M.D., Price D.D., Robinson M.E., George S.Z. (2009). The Mechanisms of Manual Therapy in the Treatment of Musculoskeletal Pain: A Comprehensive Model. Man. Ther..

[130] Meissner K., Fässler M., Rücker G., Kleijnen J., Hróbjartsson A., Schneider A., Antes G., Linde K. (2013). Differential Effectiveness of Placebo Treatments: A Systematic Review of Migraine Prophylaxis. JAMA Intern. Med..

[131] Nim C., Aspinall S., Cook C., Corrêa L., Donaldson M., Downie A., Juhl C. (2025). The Effectiveness of Spinal Manipulative Therapy in Treating Spinal Pain Does Not Depend on the Application Procedures: A Systematic Review and Network Meta-Analysis. J. Orthop. Sports Phys. Ther..

[132] Cerritelli F., Verzella M., Cicchitti L., D’alessandro G., Vanacore N. (2016). The Paradox of Sham Therapy and Placebo Effect in Osteopathy: A Systematic Review. Medicine.

[133] Paterson C., Dieppe P. (2005). Characteristic and Incidental (Placebo) Effects in Complex Interventions Such as Acupuncture. BMJ.

[134] Linde M., Fjell A., Carlsson J., Dahlöf C. (2005). Role of the Needling per Se in Acupuncture as Prophylaxis for Menstrually Related Migraine: A Randomized Placebo-Controlled Study. Cephalalgia.

[135] Diener H.-C., Kronfeld K., Boewing G., Lungenhausen M., Maier C., Molsberger A., Tegenthoff M., Trampisch H.-J., Zenz M., Meinert R. (2006). Efficacy of Acupuncture for the Prophylaxis of Migraine: A Multicentre Randomised Controlled Clinical Trial. Lancet Neurol..

[136] Tsutsumi Y., Tsujimoto Y., Tajika A., Omae K., Fujii T., Onishi A., Kataoka Y., Katsura M., Noma H., Sahker E. (2023). Proportion Attributable to Contextual Effects in General Medicine: A Meta-Epidemiological Study Based on Cochrane Reviews. BMJ Evid. Based Med..

[137] Cai L., He L. (2019). Placebo Effects and the Molecular Biological Components Involved. Gen. Psychiatry.

[138] Rossettini G., Campaci F., Bialosky J., Huysmans E., Vase L., Carlino E. (2023). The Biology of Placebo and Nocebo Effects on Experimental and Chronic Pain: State of the Art. J. Clin. Med..

[139] Peciña M., Zubieta J.-K. (2014). Molecular Mechanisms of Placebo Responses in Humans. Mol. Psychiatry.

[140] Benedetti F., Mayberg H.S., Wager T.D., Stohler C.S., Zubieta J.-K. (2005). Neurobiological Mechanisms of the Placebo Effect. J. Neurosci..

[141] Atlas L.Y., Wager T.D. (2014). A Meta-Analysis of Brain Mechanisms of Placebo Analgesia: Consistent Findings and Unanswered Questions. Handb. Exp. Pharmacol..

[142] Amanzio M., Benedetti F., Porro C.A., Palermo S., Cauda F. (2013). Activation Likelihood Estimation Meta-Analysis of Brain Correlates of Placebo Analgesia in Human Experimental Pain. Hum. Brain Mapp..

[143] Eippert F., Finsterbusch J., Bingel U., Büchel C. (2009). Direct Evidence for Spinal Cord Involvement in Placebo Analgesia. Science.

[144] Crawford L.S., Mills E.P., Hanson T., Macey P.M., Glarin R., Macefield V.G., Keay K.A., Henderson L.A. (2021). Brainstem Mechanisms of Pain Modulation: A within-Subjects 7T FMRI Study of Placebo Analgesic and Nocebo Hyperalgesic Responses. J. Neurosci..

[145] Büchel C., Geuter S., Sprenger C., Eippert F. (2014). Placebo Analgesia: A Predictive Coding Perspective. Neuron.

[146] Stein N., Sprenger C., Scholz J., Wiech K., Bingel U. (2012). White Matter Integrity of the Descending Pain Modulatory System Is Associated with Interindividual Differences in Placebo Analgesia. Pain.

[147] Lee I.-S., Wallraven C., Kong J., Chang D.-S., Lee H., Park H.-J., Chae Y. (2015). When Pain Is Not Only Pain: Inserting Needles into the Body Evokes Distinct Reward-Related Brain Responses in the Context of a Treatment. Physiol. Behav..

[148] Zunhammer M., Spisák T., Wager T.D., Bingel U.P.I.C. (2021). Meta-Analysis of Neural Systems Underlying Placebo Analgesia from Individual Participant FMRI Data. Nat. Commun..

[149] Carrasco-Uribarren A., Mamud-Meroni L., Tarcaya G.E., Jiménez-Del-Barrio S., Cabanillas-Barea S., Ceballos-Laita L. (2024). Clinical Effectiveness of Craniosacral Therapy in Patients with Headache Disorders: A Systematic Review and Meta-Analysis. Pain Manag. Nurs..

[150] Rubinstein S.M., van Middelkoop M., Assendelft W.J., de Boer M.R., van Tulder M.W. (2011). Spinal Manipulative Therapy for Chronic Low-Back Pain. Cochrane Database Syst. Rev..

